# LAMP3 is a potent uterine corpus endometrial carcinoma prognostic biomarker associated with immune behavior

**DOI:** 10.18632/aging.205414

**Published:** 2024-01-11

**Authors:** Bidong Fu, Minqin Zhou, Xitong Geng, Yike Jiang, Hong Zeng, Xuanrui Zhou, Zichuan Yu, Jingying Pan, Yanting Zhu, Hao Zheng, Shuhan Huang, Yiyang Gong, Da Huang, Yanying Zhong

**Affiliations:** 1 Department of Obstetrics and Gynecology, Second Affiliated Hospital of Nanchang University, Nanchang, China; 2 Second College of Clinical Medicine, Nanchang University, Nanchang, China; 3 Department of Thyroid Surgery, Second Affiliated Hospital of Nanchang University, Nanchang, China

**Keywords:** WGCNA, LAMP3, uterine corpus endometrial carcinoma, prognosis, immune infiltration

## Abstract

Background: Uterine corpus endometrial carcinoma (UCEC) is one of the most common gynecological malignancies and its incidence and mortality continue apace. Lysosome-associated membrane protein 3 (LAMP3) is the third member of the LAMP family and its overexpression has been described to be involved in the progression of breast, ovarian and cervical cancers, but there has been an absence of research focusing on its role in UCEC.

Methods: WGCNA, TIMER, LinkedOmics, GSEA, Cytoscape, Kaplan-Meier plotter, GDC, GeneMANIA, cBioPortal, PDB, RNAinter, miRNet were applied in this research.

Results: Our study uncovers that LAMP3 possesses higher expression levels in UCEC compared to normal tissues, and this differential expression profile is tightly aligned with clinical and pathological features, and patients demonstrating high LAMP3 expression tend to have a shorter survival expectancy. The high expression of LAMP3 is modulated by the designated ceRNA network. LAMP3 is engaged in UCEC progression by functioning in a variety of biological roles of relevance to immunity. Furthermore, we predicted several prospering drugs based on drug sensitivity. Finally, we also constructed possible docking patterns of LAMP3 with ABCA3, RAB9A, and SGTB.

Conclusions: LAMP3 is a formidable biomarker for UCEC and could be a prospective candidate for the diagnosis, treatment and prognostic assessment of UCEC.

## INTRODUCTION

Endometrial cancer (UCEC) is considered amongst the most common gynaecological malignancies, recording a 55% increase in incidence and a 23% increase in mortality over the past 30 years [1]. About 90% of cases will be coupled with abnormal uterine bleeding, but this symptom has little specificity [2]. The survival quality and expectations of patients with endometrial cancer vary depending on the time of diagnosis. When diagnosed in the early stages, the tumour is confined to the uterus and can be treated with total hysterectomy and bilateral salpingo-oophorectomy, with a five-year survival rate ranging from 74% to 91% [3]. If the diagnosis was delayed and the tumour progresses to local or distant metastases, chemotherapy would then have to be administered but the five-year survival rate would drop dramatically [4]. Frustratingly, screening tests for UCEC are not yet in place, the available biomarkers are not yet used for routine diagnosis and prognostic assessment, and confirmation of diagnosis still hinges on pathological endometrial biopsy [5, 6]. Therefore, there is an urgent need to discover more efficient biomarkers to facilitate the diagnosis, treatment and prognostic assessment of UCEC.

Lysosomal-associated membrane protein 3 (LAMP3) is the third member of the LAMP family of proteins, whose coding gene is located on chromosome 3q27.1 and was originally reported to be specifically expressed in lung tissue [7]. In the same year, LAMP3 was also identified as a molecular marker for mature dendritic cells, hence earning the name DC-LAMP [8]. LAMP3 has been reported to have profound significance in the progression of a variety of tumours such as oral squamous cell carcinoma [9], oesophageal squamous cell carcinoma [10], gastrointestinal carcinoma [11], hepatocellular carcinoma [12], not to mention common gynaecological tumours such as breast [13], ovarian [14] and cervical cancers [15]. For example, overexpression of LAMP3 is able to boost the metastasis of cervical cancer cell lines *in vivo* and *in vitro*. Alternatively, it can mediate hypoxia-induced migration of breast cancer cells [16]. However, the role of LAMP3 in endometrial cancer has been overlooked by previous researchers.

Within this research, we screened for LAMP3 by the WGCNA approach in combination with gene characterisation and explored the differential expression of LAMP3 in endometrial cancer and normal tissues, the relationship with clinicopathological features, and the mechanisms of expression regulation. The impact of its differential expression on patients’ survival expectations was followed by analysis. Subsequently, the biological functions of LAMP3 were analysed and the immune-related mechanisms of LAMP3 in UCEC were explored in depth. Several potential drugs were identified through drug sensitivity analysis. Lastly, we evaluated the protein interaction network of LAMP3 and selected several proteins to predict their potential docking patterns with LAMP3.

## MATERIALS AND METHODS

### Patients and tumor specimens

We collected UCEC tissues and matched non-tumor tissues from a cohort of 20 patients at the Second Affiliated Hospital of Nanchang University between January 2020 and January 2022. These samples were promptly frozen in liquid nitrogen and stored at -80° C. The study was carried out with the informed consent of the patients and received approval from the Research Ethics Committee of the Second Affiliated Hospital of Nanchang University.

### Data source and processing

Our data were mainly collected from The Cancer Genome Atlas (TCGA) database (https://cancergenome.nih.gov). From this, we gained RNAseq data in FPKM format for the STAR workflow of UCEC tumor and used it to comprehensively characterize the expression of LAMP3 in UCEC, the prognosis of UCEC patients with high LAMP3 expression, and the relationship of LAMP3 expression with several clinic features, diverse signaling pathways, immune infiltration, immune checkpoints and RNA-modified genes.

### Weighted gene co-expression network analysis (WGCNA)

WGCNA is a bioinformatics analytical approach for seeking modules of highly relevant genes [17]. In this research, we processed all differentially expressed genes in TCGA-UCEC by R (4.1.2) package “WGCNA”, applying a soft threshold function to compute power parameter, generating a cluster dendrogram with hierarchical clustering, structuring a module-trait relationship network by assigning normal and tumor samples to 0, 1 and showing the association between gene significance and module membership in the yellow module with the scatter plot.

### GEPIA website

GEPIA (http://gepia.cancer-pku.cn/) is a platform serving us with RNA sequencing expression data in TCGA and GTEx databases [18]. On the basis of TCGA data, we studied the differential expression of LAMP3 in UCEC and normal tissues utilizing the “Boxplot” section in the “Expression DIY” module.

### TIMER database

TIMER (https://cistrome.shinyapps.io/timer/) gives us a user-friendly network interface for exploring tumor-immune interactions [19]. By this way, we characterized the expression of LAMP3 in all TCGA cancers.

### CCLE database

Cancer Cell Line Encyclopedia database (https://portals.broadinstitute.org/ccle/about) produces a massive genomic dataset with 947 human cancer cell lines [20]. We acquired cell line gene expression data from it for the purpose of assessing LAMP3 expression level in diverse cancers.

### Cell culture

Transfection of the human UCEC cell line TCHu198 in 37° C DMEM (HyClone, Germany) and 10% female bovine serum (Gibco, USA).

### Kaplan–Meier plotter database

The Kaplan-Meier plotter database (http://kmplot.com) is a survival analysis tool which allows the assessment for disease progression [21]. Through “mRNA RNA-seq” module, we examined the prognostic value of LAMP3 in UCEC, the differential survivorship of UCEC patients with a variety of immune cell subtypes and the survival curves of 4 RNA modification-related genes in UCEC.

### LinkedOmics database analysis

LinkedOmics (http://linkedomics.org/login.php) presents us with a unique podium covering multi-omics data for 32 types of cancers [22]. With RNAseq data supplied by the HiseqRNA platform in the TCGA-UCEC cohort, we investigated the co-expressed genes of LAMP3 and their enrichment pathways.

### Gene set enrichment analysis (GSEA) analysis

GSEA is a powerful profiling approach which uncovers the common biological pathways in a group of genes by focusing on the gene set [23]. In our study, we refer to the “c2, cp.kegg.v2022.1.Hs, symbols.gmt” gene set and include the three requirements of FDR < 0.25, P-value < 0.05 and false discovery rate (FDR) > 1 in the filtration criteria to perform an enrichment assessment of the LAMP3 high expression group.

### Protein-protein interaction (PPI) network analysis

The STRING database (https://string-db.org) allows for exploration of genomic links among genes encoding proteins, thus we can infer the biological functions of proteins [24]. Our study utilized the STRING database to research the first 500 co-expressed genes of LAMP3 and set a medium confidence level = 0.9 to select genes, and then we created an overall PPI network by visualizing them with Cytoscape software. What’s more, the most essential module of our PPI network was constructed by Cytoscape Molecular Complex Detection (MCODE) plug-in. The relevant parameters are set as follows: Max depth=100, node score cutoff=0.2, K-core=2.

### TISIDB analysis

TISIDB database (http://cis.hku.hk/TISIDB/) could be employed to inquire the relationship of specific genes with immunity in the context of tumorigenesis [25]. Through the “Overall survival” section in the “Clinical” module, we probed the prognostic value of LAMP3 in UCEC; through the “Immunomodulator” and “Chemokine” modules, we sought the relationship between LAMP3 expression and cytokines in UCEC, and by “Subtype” module, we looked into the distribution of LAMP3 expression in different immune subtypes in UCEC.

### Comparative toxicogenomics database

The CTD (http://ctdbase.org/) integrates the interactions of specific genes with correlative compounds and contains the literature coverage of some compounds [26]. From it, we obtained several drugs regulating LAMP3 expression.

### Protein structure and docking analysis

The cBioPortal database (https://www.cbioportal.org/) incorporates multidimensional cancer genomics data [27]. Through this database, we built the secondary structures of some proteins (LAMP3, ABCA3, SGTB and RAB9A) with Uterine Corpus Endometrial Carcinoma (TCGA, Firehose Legacy). The PDB database (https://www.rcsb.org/) stores experimentally determined 3D structures of biomolecules and their complexes [28]. It was adopted for downloading the tertiary spatial conformation of 4 proteins. Moreover, the HDOCK server (http://hdock.phys.hust.edu.cn/) and PyMOL software were respectively applied to predict and visualize the docking models of LAMP3-ABCA3, LAMP3-SGTB and LAMP3-RAB9A.

### GeneMANIA analysis

GeneMANIA (https://genemania.org/) database delivers genomics and proteomics data for seeking genes functionally similar to a given gene. By it, we constructed a LAMP3-centered interaction network.

### ceRNA analysis

By TargetScan (http://www.targetscan.org), DIANA (http://diana.imis.athena-innovation.gr/DianaTools/index) and RNAinter (http://www.rnainter.org) online sites, we jointly predicted miRNAs targeting LAMP3 and screened for miRNAs negatively correlated with LAMP3 in the TCGA-UCEC cohort.

Besides, by miRNet2.0 (https://www.mirnet.ca/miRNet/home.xhtml) and starBase3.0 (https://rnasysu.com/encori/) online sites, we searched for targeted lncRNAs of has-miR-106a-5p and has-miR-499a-5p, and again utilized the starBase3.0 tool to select lncRNAs that met the negative correlation condition with the corresponding miRNAs. And then, we also exploited Sankey diagrams to build LAMP3-related ceRNA networks.

### Quantitative real-time PCR (qRT-PCR)

We followed the Trizolrogen (USA) method to extract total RNA and then used an RT-PCR kit to convert the extracted RNA into complementary DNA (cDNA). To assess the mRNA levels of the target genes, a quantitative real-time PCR kit was used.

### Western blot

We utilized radioimmunoprecipitation assay buffer (RIPA) with the addition of protease inhibitor cocktail (Roche, Switzerland) to extract total proteins and quantified using the BCA method. Protein was separated on SDS-PAGE gels and transferred to PVDF membranes (EMD Millipore, Billerica, MA, USA). After overnight incubation at 4° C with the designated antibodies, the membrane was subjected to three washes with TBST. The membrane was then exposed to secondary antibodies at room temperature for one hour. Detection of protein expression was conducted using the Amersham™ ImageQuant 800 system (GE Healthcare, USA).

### Transwell assay

Invasion assays were performed using 24-well transwell chambers with a Matrigel precoated upper chamber. Each group cell suspension was added to a Matrigel precoated chamber, with the lower chamber containing a higher serum concentration than the upper chamber. The samples were then incubated for 24 hours in a cell culture incubator. Following incubation, the cells were fixed with methanol and stained using hematoxylin-eosin.

### Statistical analysis

Our research utilized R for all data analysis (3.6.3/4.0.3/4.1.2). With the R package “ggplot2”, we predicted the expression of LAMP3 in normal and UCEC tissues applying the Wilcoxon rank sum test, and the association between LAMP3 and lots of clinical features was estimated by the Kruskal-Wallis test. Furthermore, this package was also been employed to infer the linkage of RNA-modified genes with LAMP3 expression and the correlation between some molecules. Alternatively, based on the “survminer” package, the “survival” package and the “rms” package, we evaluated the effect of LAMP3 on the survivorship of UCEC patients and the accuracy of the column line plot, while the ROC curve was resolved and visualized with the “timeROC” package and the “ggplot2” package. Besides, by “GSVA” package, we also selected the parameter method=‘ssgsea’ to calculate the correlation between LAMP3 and pathway score; and the “ssGSEA” method was applied for finding out the relationship between LAMP3 and immune cell infiltration. In parallel, the “estimate” package was chosen for the characterization of the association of LAMP3 expression with 3 types of scores as well as immune checkpoint genes.

### Data availability statement

The datasets used and/or analyzed during the current study are available from the corresponding author on reasonable request.

## RESULTS

### Construction of a weighted co-expression network in UCEC

**Figure 1:**
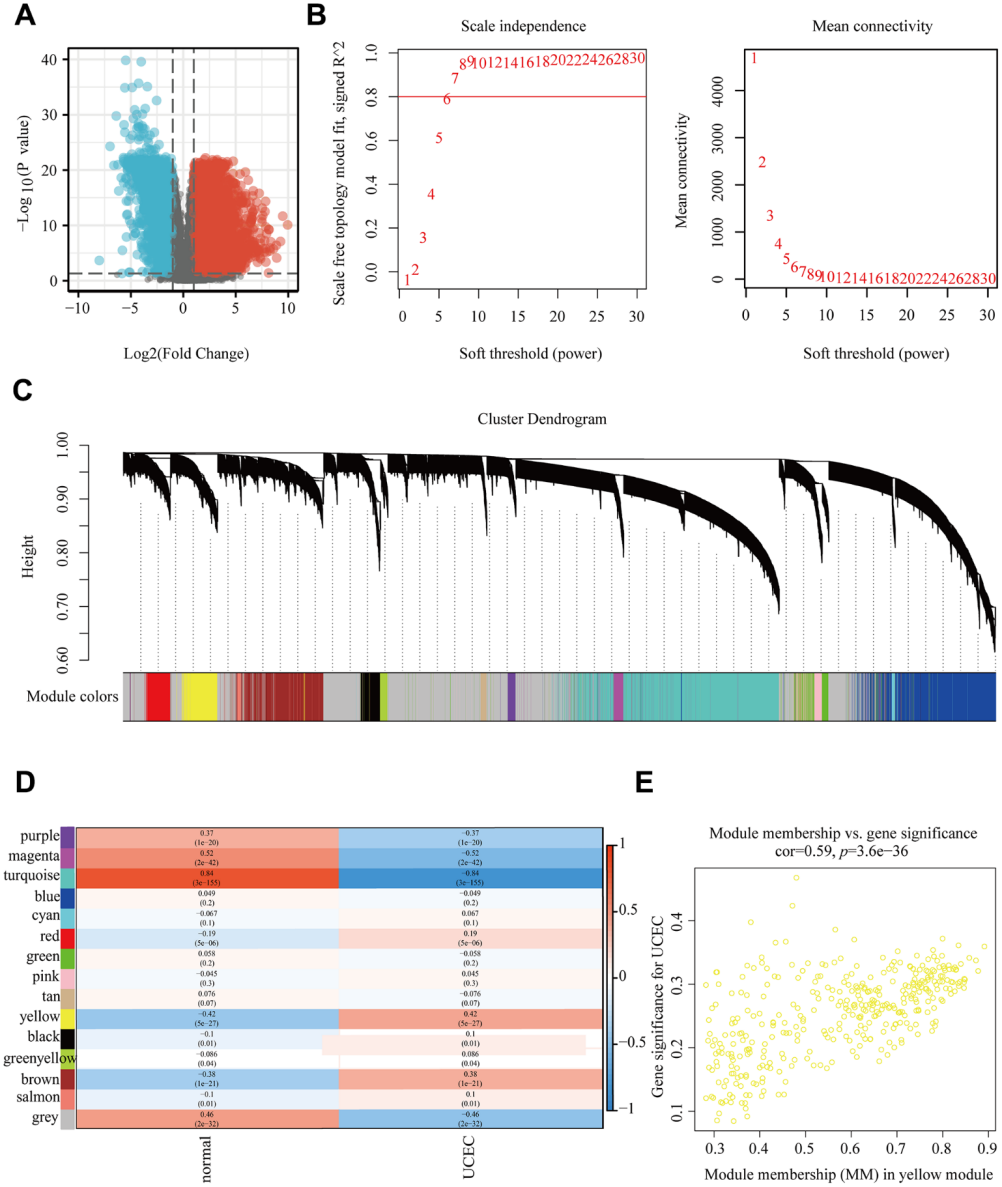
**WGCNA based on TCGA-UCEC.** (**A**) All differentially expressed genes in TCGA-UCEC. (**B**) Determination of soft threshold. (**C**) Tree diagram of 15 modules labeled with different colors. (**D**) Correlation between module feature genes and clinical traits. (**E**) Scatter plot of MM vs GS in the yellow module.

For identifying the key gene module of UCEC, we performed a WGCNA based on the values of gene expression. We first examined 26535 genes in the TCGA-UCEC dataset and detected 9037 genes from it that exhibited differential expression (Figure 1A). Subsequently, according to the scale independence and average connectivity, we set the soft threshold to 6 (R2= 0.8) (Figure 1B). And 15 modules were identified with dynamic number cutting package by setting 30 as the minimum number of genes per genetic network and 0.25 as the clustering height limit (Figure 1C). Moreover, we evaluated the correlation between modules and UCEC by investigating the relevance of ME values to UCEC samples, which revealed that yellow module was notably associated with UCEC (r=0.42, p=5e-27) (Figure 1D). Furthermore, the MM vs GS analysis also demonstrated that genes in the yellow module were highly associated with UCEC (Figure 1E).

In the next step, by performing survival, univariate and 1.3.5-year ROC analyses on 371 genes in the yellow module successively, we filtered out 73 genes in which survival, univariate values were significant (p<0.05) and 1,3,5-year ROC values were greater than 0.5. Then, to further tighten the constraints, we ranked these 73 genes depending on the HR values in the univariate analysis and picked out the top 30 to review the literature, discovering that only 6 of them had not yet been reported in UCEC, which were KLRG2, C5orf34, CCDC150, TDRKH, LAMP3, KLC3 (Table 1). To sum up, it is suggested that LAMP3 may be one of the multiple factors that influence the development of UCEC.

**Table 1:** Univariate analysis of 6 genes in UCEC.

**Gene name**	**Gene id**	**Gene biotype**	**HR**	**CI**	**p.Cox**
KLRG2	ENSG00000188883	protein coding	2.866	1.812-4.533	<0.001
C5orf34	ENSG00000172244	protein coding	2.319	1.502-3.582	<0.001
CCDC150	ENSG00000144395	protein coding	2.095	1.362-3.221	<0.001
TDRKH	ENSG00000182134	protein coding	2.023	1.324-3.092	0.001
LAMP3	ENSG00000078081	protein coding	1.975	1.295-3.012	0.002
KLC3	ENSG00000104892	protein coding	1.962	1.284-2.998	0.002

HR, hazard ratio; CI, confidence interval.

### mRNA expression of LAMP3 in UCEC

**Figure 2:**
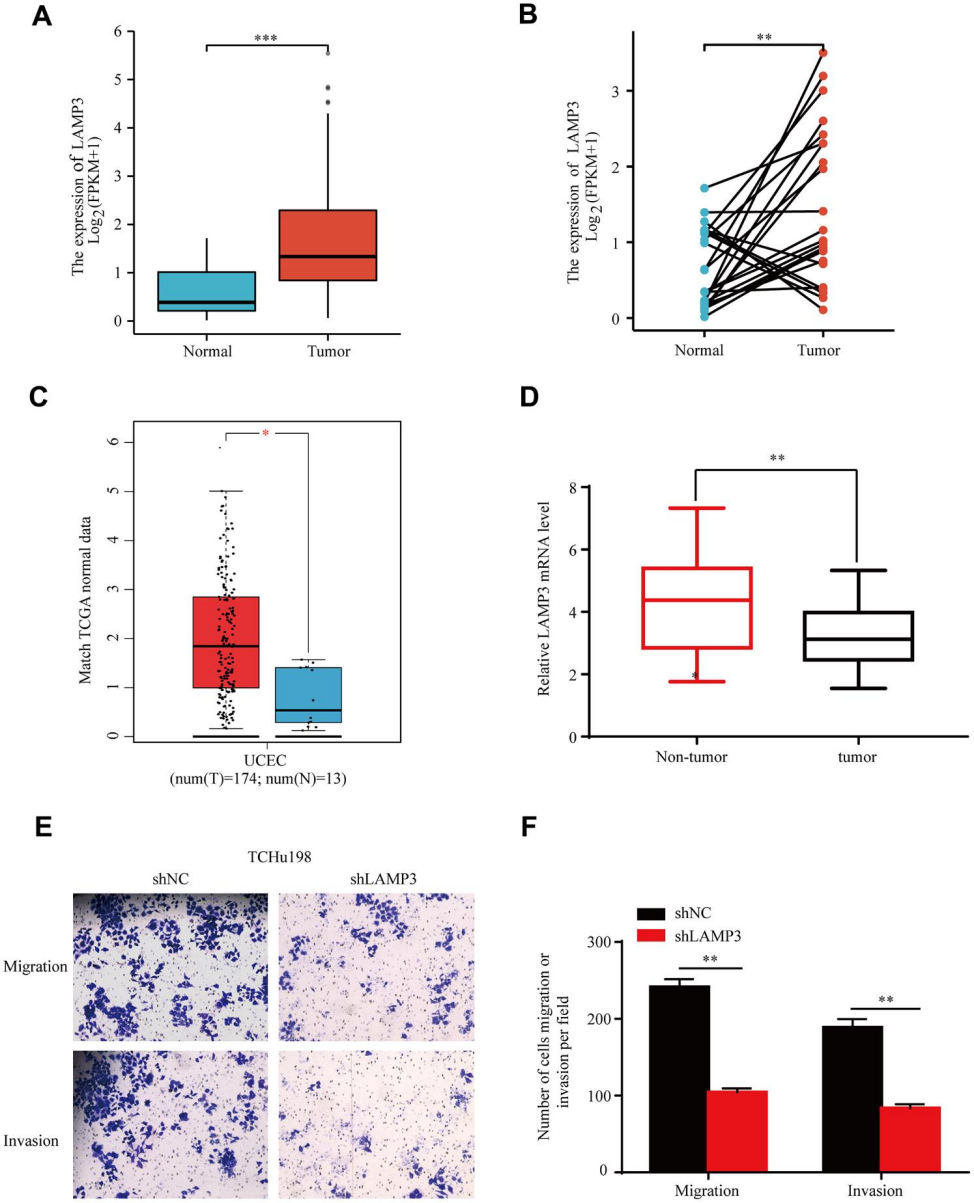
**Overexpression of LAMP3 in UCEC tissues.** Expression of LAMP3 in UCEC tissues and normal tissues presented by TCGA data in the form of (**A**) scatter plot and (**B**) paired difference plot. (**C**) mRNA transcript level of LAMP3 in UCEC gained by GEPIA database. (**D**) Quantification of LAMP3 mRNA expression based on qRT-PCR. (**E**, **F**) Migration and invasion ability of TCHu198 cells in the shNC and shLAMP3 groups.

For the sake of determining LAMP3 expression in UCEC, we investigated the transcript level of LAMP3 through a series of databases. With the CCLE database, we explored the expression distribution of LAMP3 in 32 tumor tissues, and the outcome implied that it possessed high expression in various tumor tissues such as SCLC, BLCA, ESCA, and UCEC (Supplementary Figure 1A). Then, we further resolved LAMP3 expression in tumor and normal tissues utilizing RNA-seq data from the TIMER website, identifying that LAMP3 exhibited higher expression in 11 types of tumor tissues, including UCEC (Supplementary Figure 1B). Subsequently, we probed the level of LAMP3 expression in single tumor UCEC using the collected UCEC tissues and normal tissues from the TCGA database, which suggested an upregulation of LAMP3 expression in UCEC tissues (Figure 2A, 2B). Also, the above result was validated on the basis of the GEPIA database, as illustrated in Figure 2C, it remained in line with our expectation. In addition, we discovered that the expression of LAMP3 was upregulated through qRT-PCR (Figure 2D). And to further elucidate the impact of LAMP3 expression on the metastasis of UCEC cells, we conducted transwell assays. Impressively, our findings revealed that the suppression of LAMP3 significantly impeded the migratory and invasive capabilities of TCHu198 cells (Figure 2E, 2F). In summary, we conclude that LAMP3 is overexpressed in UCEC tissues when compared to normal tissues.

### Association of LAMP3 expression with multiple clinicopathological features in UCEC

With the purpose of further exploring the association of LAMP3 with some clinical characteristics in UCEC, we compared the level of LAMP3 expression in UCEC patients with distinct clinicopathological characteristics based on the TCGA-UCEC data. According to the age group, it could be noted that LAMP3 expression was upregulated in UCEC patients older than 60 years (Supplementary Figure 2A). Concerning the grade and stage group, we observed that Grade 3 and Stage IV UCEC patients possessed higher expression compared to normal people and early grades or early stages UCEC patients (Supplementary Figure 2B, 2C). In terms of histological type, the expression of LAMP3 in Endometrioid, mixed, and serous UCEC patients were all upregulated compared to the normal group, and the serous UCEC patients displayed higher expression in contrast to Endometrioid UCEC patients (Supplementary Figure 2D). Moreover, on the survival status, the dead patients had apparently higher LAMP3 expression (Supplementary Figure 2E). Also, it was detected that LAMP3 expression was correlated with menopausal status with varying level of LAMP3 expression at different periods, while all periods were higher than normal (Supplementary Figure 2F). In conclusion, it is indicated that LAMP3 expression is strongly related to various clinicopathological features.

### LAMP3 may be a potential prognostic marker for UCEC

**Figure 3:**
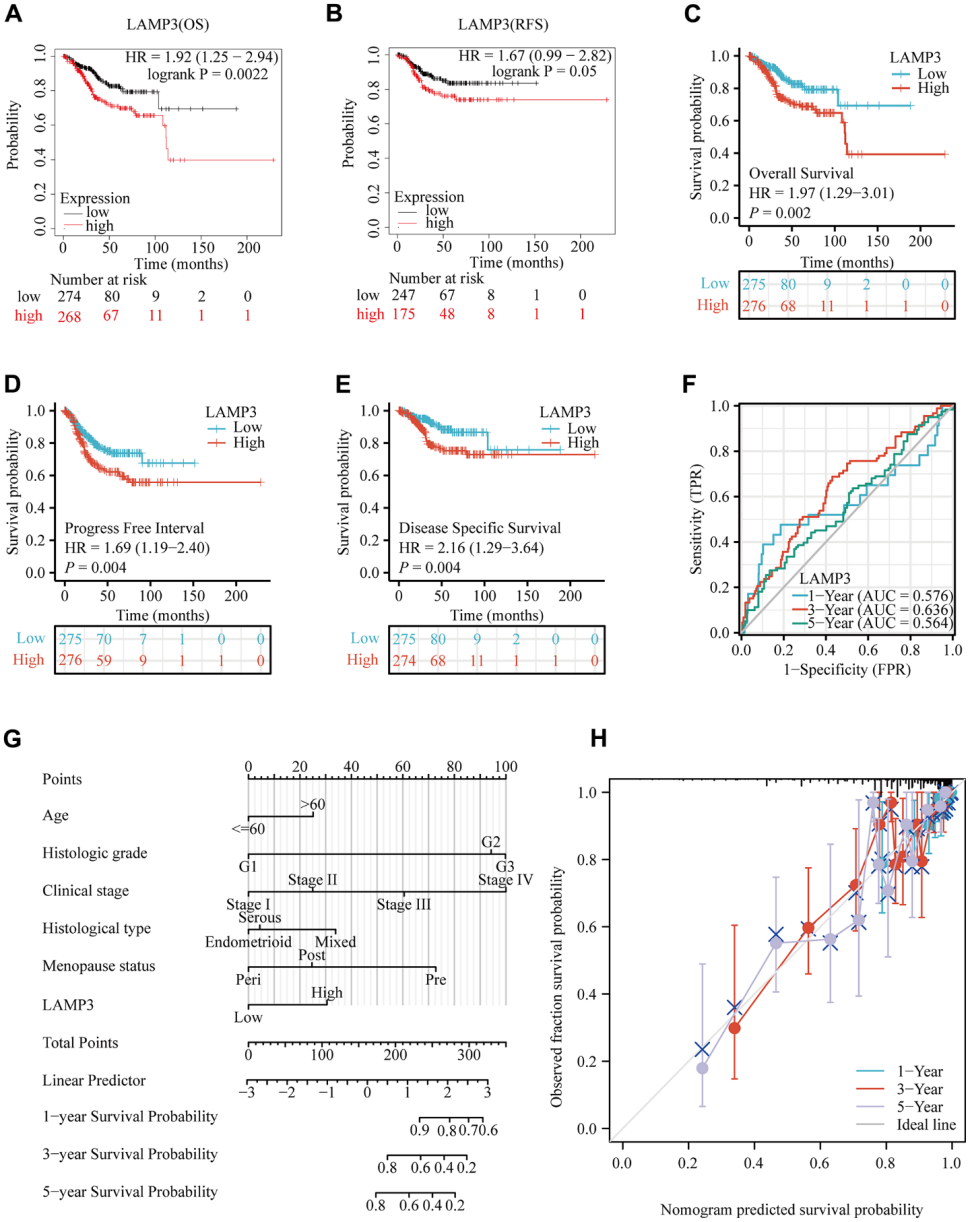
**Prognostic value of LAMP3 in UCEC patients.** (**A**, **B**) Demonstration of the relationship between LAMP3 expression and survival of UCEC patients on the basis of Kaplan-Meier plotter database. (**C**) Overall survival, (**D**) Progress free interval, (**E**) Disease specific survival of LAMP3 in UCEC with TCGA data. (**F**) A time-independent ROC curve demonstrates the predictive power of LAMP3. (**G**) A column line graph presents the impact of clinical features and LAMP3 expression on the prognosis of UCEC patients. (**H**) Calibration curve of the column line graph.

Aiming to measure the prognostic potential of LAMP3 in UCEC, we probed the influence of LAMP3 expression on the survivorship of UCEC sufferers with multiple methods. Initially, we adopted Kaplan-Meier plotter database to plot survival curves for determining the correlation of LAMP3 expression with the survival of UCEC sufferers. The outcome Figures 3A, 3B demonstrated that patients with high expression of LAMP3 in UCEC possessed worse OS and RFS. Later on, in a further survival analysis of UCEC patients, we separated LAMP3 expression into high and low expression groups through TCGA data, and it was concluded that high LAMP3 expression group owned worse OS, PFI and DSS in comparison with the low LAMP3 expression group (Figure 3C–3E). A time-dependent ROC curve demonstrated the favorable predictive capability of LAMP3 for survival of UCEC patients (Figure 3F). Alternatively, the column line graph was constructed based on various clinicopathological features, which evidenced that Histologic grade and Clinical stage contributed the most to the prognosis of UCEC patients, and LAMP3 high expression was indeed remarkably related to a low survival probability of UCEC sufferers (Figure 3G). The corresponding calibration plot exhibited excellent agreement with the column plot for the 1, 3, 5 years forecasted (Figure 3H). In summary, high LAMP3 expression is considered as a predictor of adverse outcome in UCEC patients.

### Co-expression patterns of LAMP3 in UCEC

**Figure 4:**
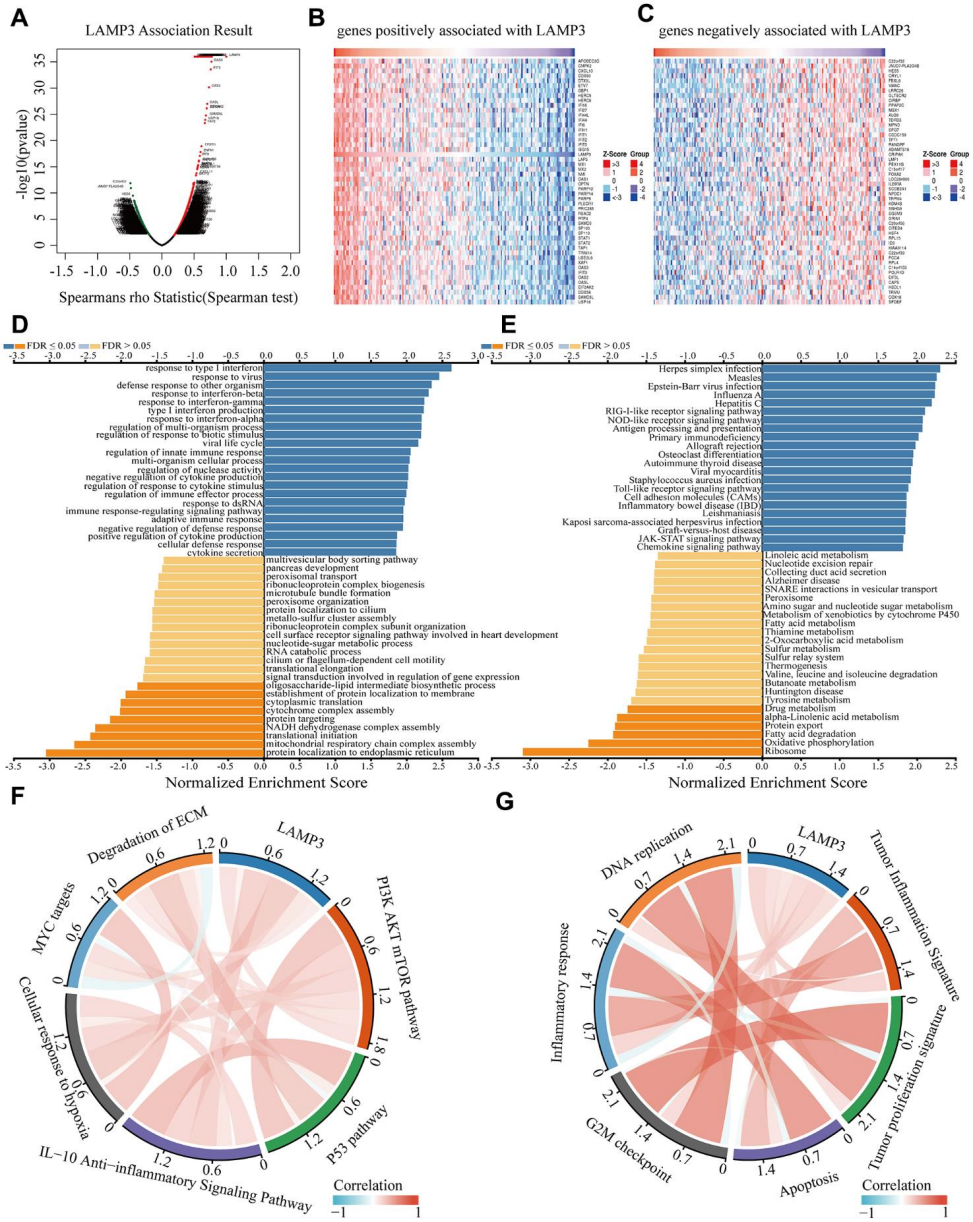
**Construction of co-expression network of LAMP3.** (**A**) A spearman test is used to examine all co-expressed genes of LAMP3. The heatmap shows the top 50 genes that are (**B**) positively and (**C**) negatively associated with LAMP3. (**D**) Go analysis (biological process) and (**E**) KEGG analysis reveal the signaling pathways of LAMP3 and its co-expressed genes in UCEC by LinkedOmics. (**F**, **G**) Association between LAMP3 and each pathway by TCGA data.

Trying to gain an understanding of the biological meaning of LAMP3 in UCEC, we dug deeper into its co-expression pattern in UCEC. With the “LinkFinder” module in Linkedomics, we had detected 19898 possibly co-expressed genes with LAMP3, of which 4408 genes (red dots) were positively related and 2559 genes (green dots) were reversed (p < 0.05) (Figure 4A). Figure 4B, 4C presented the top 50 genes showing the most positive and negative association with LAMP3, respectively. Besides, via the “Linkinterpreter” module, we carried out biological process and molecular functional analysis of these genes. Go analysis (biological process) illustrated that LAMP3 co-expressed genes were primarily involved in signaling pathways in response to various interferons, defense responses to other organisms and regulation of innate immune response, while inhibitory pathways such as translational initiation (Figure 4D). KEGG pathway indicated that these genes were predominantly abundant in herpes simplex infection, NOD-like receptor signaling pathway, antigen processing and presentation, Toll-like receptor signaling pathway, chemokine signaling pathway and other pathways (Figure 4E). Additionally, with the aid of validation by RNAseq data of UCEC extracted from the TCGA database, we likewise observed that LAMP3 was extensively involved in some pathways such as G2M checkpoint and Tumor proliferation signature (Figure 4F, 4G).

Apart from that, in some further GSEA analysis, we classified all samples into high and low expression groups based on the expression level of LAMP3, where we noticed that LAMP3 high expression group was mainly enriched in typical immune-related pathways such as antigen processing and presentation, B-cell receptor signaling pathway. Together, these results propose that LAMP3 may exert a broad impact on the UCEC transcriptome through immune and pathogen-associated pathways (Supplementary Figure 3A–3K).

### LAMP3 relevant PPI network in UCEC

**Figure 5:**
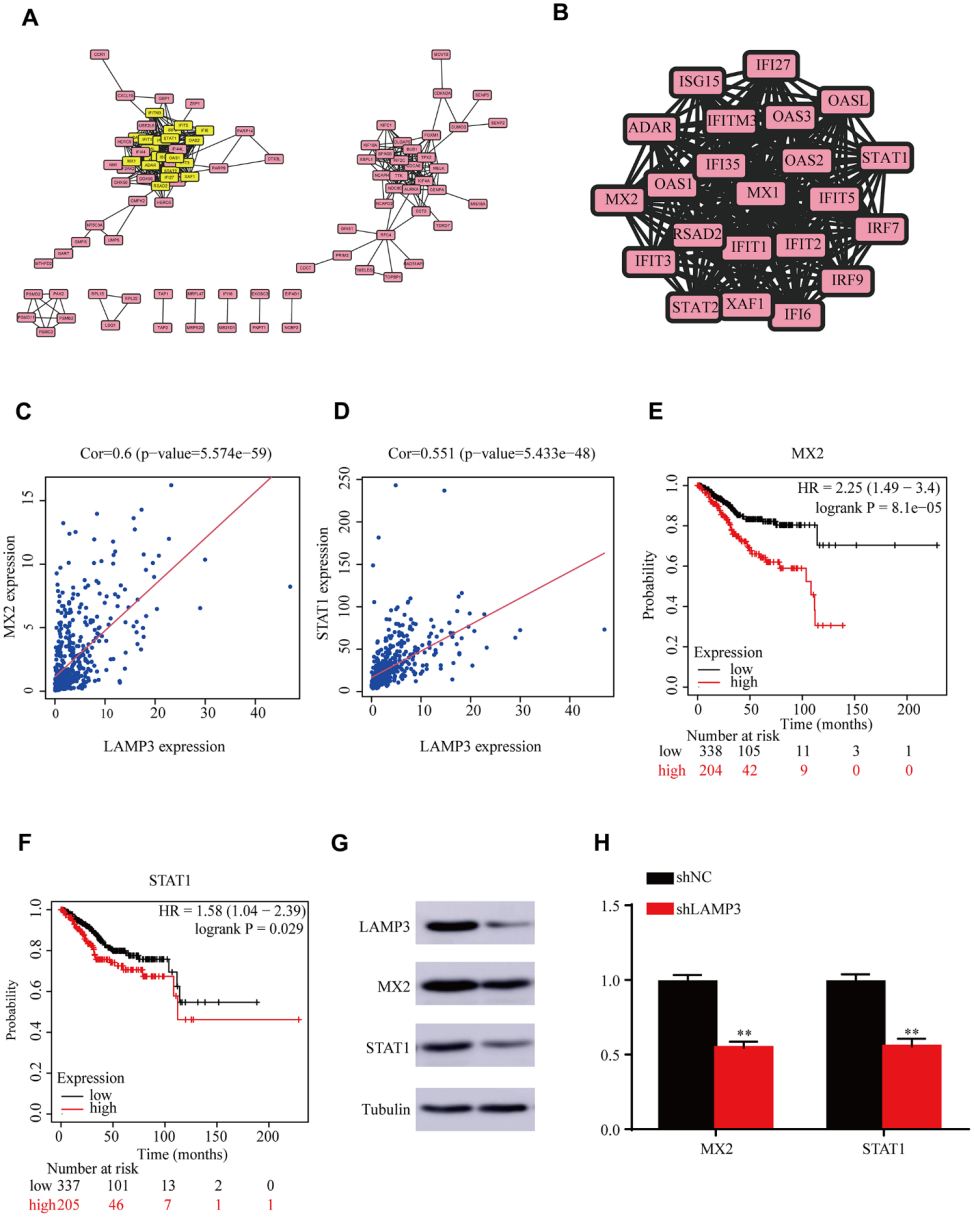
**PPI network of LAMP3 co-expressed genes.** (**A**) Protein-protein interaction (PPI) network of the first 500 co-expressed genes of LAMP3. (**B**) The most important module of protein-protein interaction network. (**C**, **D**) Correlation of LAMP3 with MX2 and STAT1. (**E**, **F**) Effect of MX2 and STAT1 on the prognosis of UCEC patients. (**G**) Expression of 3 genes in UCEC cells detected by western blot. (**H**) Expression of MX2 and STAT1 in shNC and shLAMP3 groups detected by qRT-PCR.

For further inferring the potential biological functions of LAMP3, we investigated the top500 co-expressed genes of LAMP3 by STRING database and established their PPI network (Figure 5A). Rating based on degree of connectivity, we also constructed the module in which LAMP3 was the most relevant and had the highest average connectivity score (21.619) by use of Cellular Landscape (MCODE plugin). As result shown in Figure 5B, among them, a total of 22 genes were included and highlighted in yellow. It was worth noting that by searching the relevant literature for these genes, we identified that all 22 genes were strongly associated with immunity. Later, we further selected the two highest scoring genes (MX2 and STAT1) for a follow-up study. As Figure 5C, 5D indicated in the scatter plots, it could be noted that MX2 and STAT1 did have a strong correlation with LAMP3; as displayed in survival curves Figure 5E, 5F, we could discover that UCEC patients with high MX2 or STAT1 expression had a poorer prognosis. Furthermore, we conducted additional experimental validation to establish the correlation between LAMP3 and the aforementioned genes. We employed western blot and qRT-PCR to investigate the expression level of MX2 and STAT1 in shNC and shLAMP3 groups, respectively. Notably, our results demonstrated a significant reduction in the expression of both genes, observed at both the protein and mRNA levels, following the inhibition of LAMP3 (Figure 5G, 5H). Therefore, we speculate that the influence of LAMP3 on the survivorship of UCEC sufferers may correlate with immunity.

### Association between LAMP3 expression and immune infiltration in UCEC

**Figure 6:**
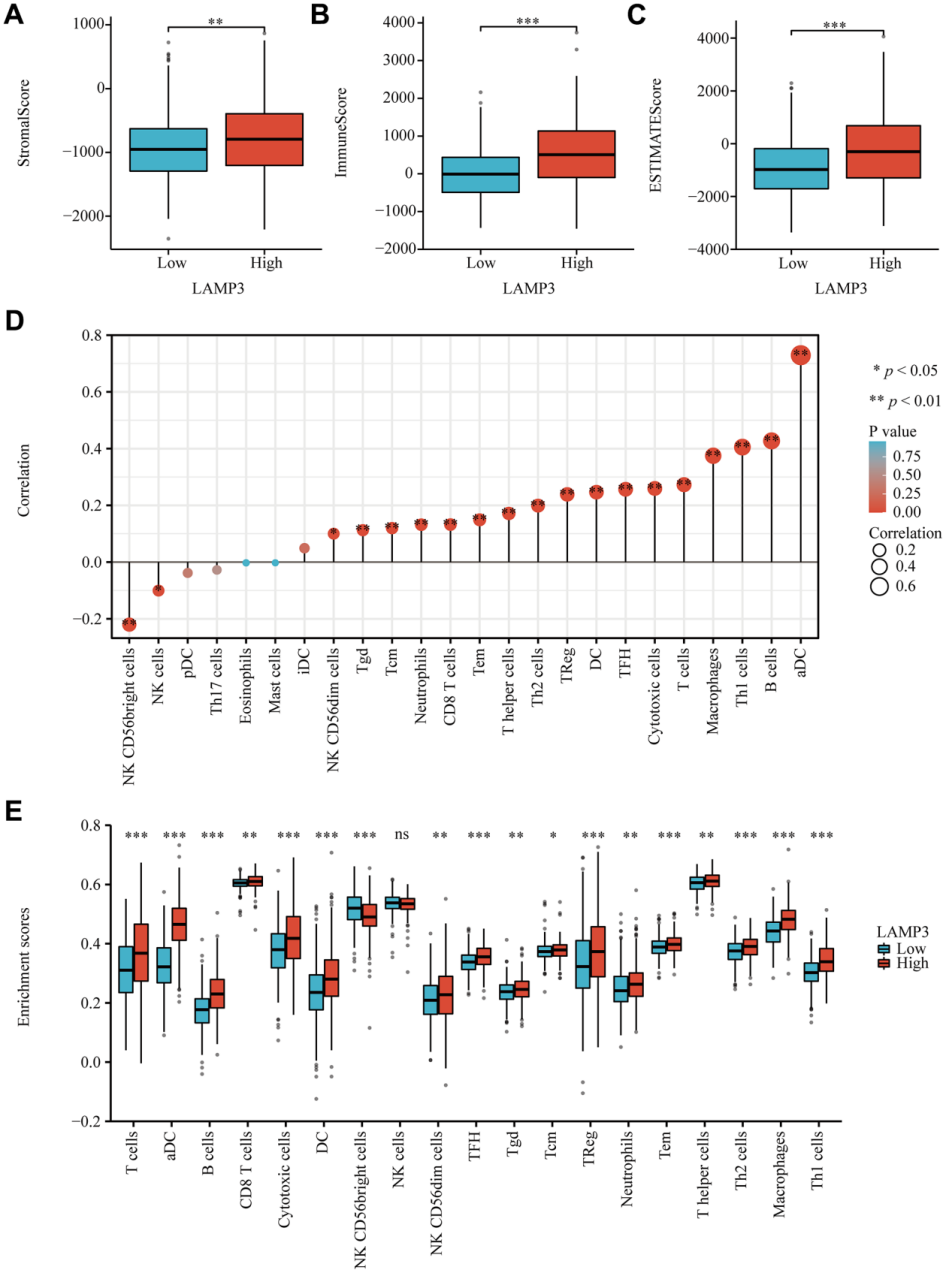
**Association of LAMP3 with immune cell infiltration in UCEC.** (**A**–**C**) Box plots display the StromalScore, ImmuneScore and ESTIMATEScore of LAMP3 in UCEC based on the “Estimate” algorithm. (**D**) Lollipop plot exhibits the correlation between LAMP3 expression and 24 immune cells. (**E**) Grouped comparison plot presents the enrichment score of 19 immune cells between the two groups with high and low expression of LAMP3.

In order to fully address the immune-related features of LAMP3, its association with immune infiltration was examined. The first step was to separate the tumor samples into two groups by the high and low expression of LAMP3, and then we calculated the StromalScore, ImmuneScore and ESTIMATEScore for each tumor sample, finding that all three scores were better in the LAMP3 high expression group compared to the LAMP3 low expression group (Figure 6A–6C). Next, with the RNAseq data from TCGA, we studied the correlation of LAMP3 expression with 24 immune cells in UCEC, as depicted in Figure 6D, 19 of these immune cells were affected by LAMP3 expression, of which it was strongly linked to the infiltration of aDC cells (r=0.729, p<0.01). Moreover, we measured the enrichment scores of these 19 immune cells in UCEC within the high and low expression groups of LAMP3, and we discovered that there were remarkable differences between the groups of 18 types of immune cells (Figure 6E).

Next, with an aim to broaden the insight of the relevance of LAMP3 with the immune infiltration, we also evaluated the association between LAMP3 expression and a variety of cytokines as well as the expression of LAMP3 in distinct immune subgroups of UCEC patients through the TISIDB database. Supplementary Figure 4A–4C displayed a positive relation of LAMP3 expression with most MHCs, chemokines and receptors in UCEC, where the 8 chemokines most correlated with LAMP3 were plotted in the scatter plots (Supplementary Figure 4D–4K). As for Supplementary Figure 4L, it revealed that LAMP3 expression was highest in the C2 subgroup (IFN-gamma dominant) and lowest in the C1 subgroup (wound healing) among the five immune subgroups. The above-mentioned outcomes imply that LAMP3 has a close connection with immune cell infiltration in UCEC.

### Linkage between LAMP3 and immune checkpoints in UCEC

Previous researches have shown that immune checkpoints have a vital function in the human immune system. Therefore, by extracting data downloaded from TCGA, we inquired into the relation between LAMP3 expression and immune checkpoints, and the result indicated that there exists a relevance between LAMP3 expression and the expression of most checkpoint genes in UCEC (Supplementary Figure 5A). Besides, the immunostimulant CD274, IL12A and immunosuppressants C10orf54, CXCL10, ICAM1, BTN3A1 were the six checkpoint genes with the highest correlation (Supplementary Figure 5B–5G). With these results, it is proposed that LAMP3 may be involved in the overexpression of checkpoint genes.

### The function of LAMP3 on the prognosis of UCEC patients may be tied to immunity

**Figure 7:**
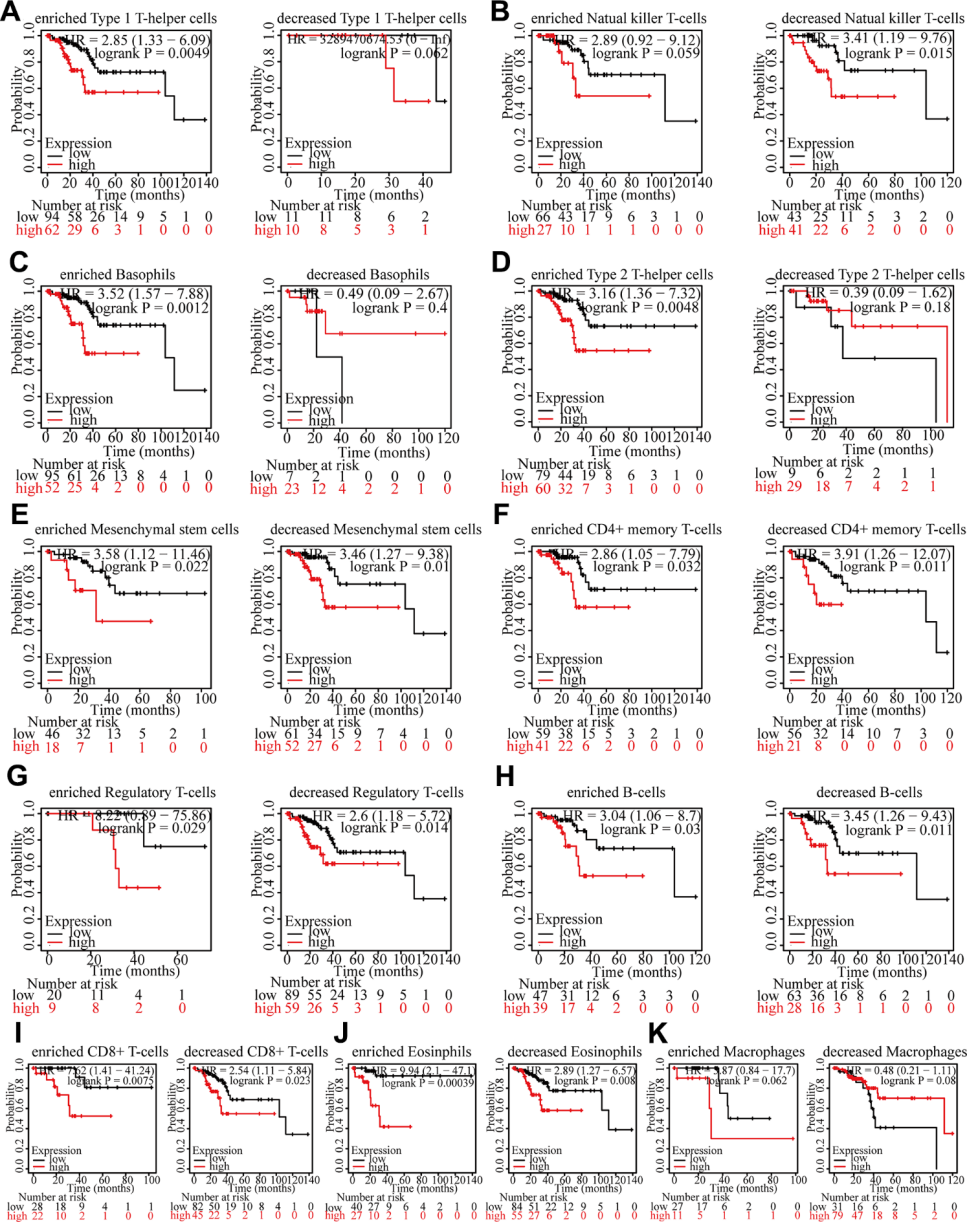
**Survival curves of high and low expression of LAMP3 in UCEC in diverse immune cell subgroups.** (**A**) Type1 T-helper cells (**B**) Natural killer T-cells (**C**) Basophils (**D**) Type2 T-helper cells (**E**) Mesenchymal stem cells (**F**) CD4+ memory T-cells (**G**) Regulatory T-cells (**H**) B-cells (**I**) CD8+ T-cells (**J**) Eosinophils (**K**) Macrophages.

Since LAMP3 expression is relevant to the immune infiltration and poor prognosis of UCEC patients respectively, we studied deeper whether LAMP3 could influence the survival of UCEC sufferers by immune infiltration. In UCEC, we carried out survival analyses on the ground of LAMP3 expression in various immune cell subsets through Kaplan-Meier plotter database (Figure 7A–7K). As the findings demonstrated, high expression of LAMP3 in the Type 1 T-helper cells enriched, Natural killer T-cells decreased, Basophils enriched, and Type 2 T-helper cells enriched cohorts resulted in poor prognosis, but in the Type 1 T-helper cells decreased, Natural killer T-cells enriched, Basophils decreased, and Type 2 T-helper cells decreased cohorts, high/low expression of LAMP3 did not affect the prognosis of UCEC patients. This result suggests that the pathway by which LAMP3 expression affects the survival of UCEC patients may have an association with the infiltration of Type 1 T-helper cells, Natural killer T-cells, Basophils and Type 2 T-helper cells.

### LAMP3 may be tied with RNA modifications in UCEC

**Figure 8:**
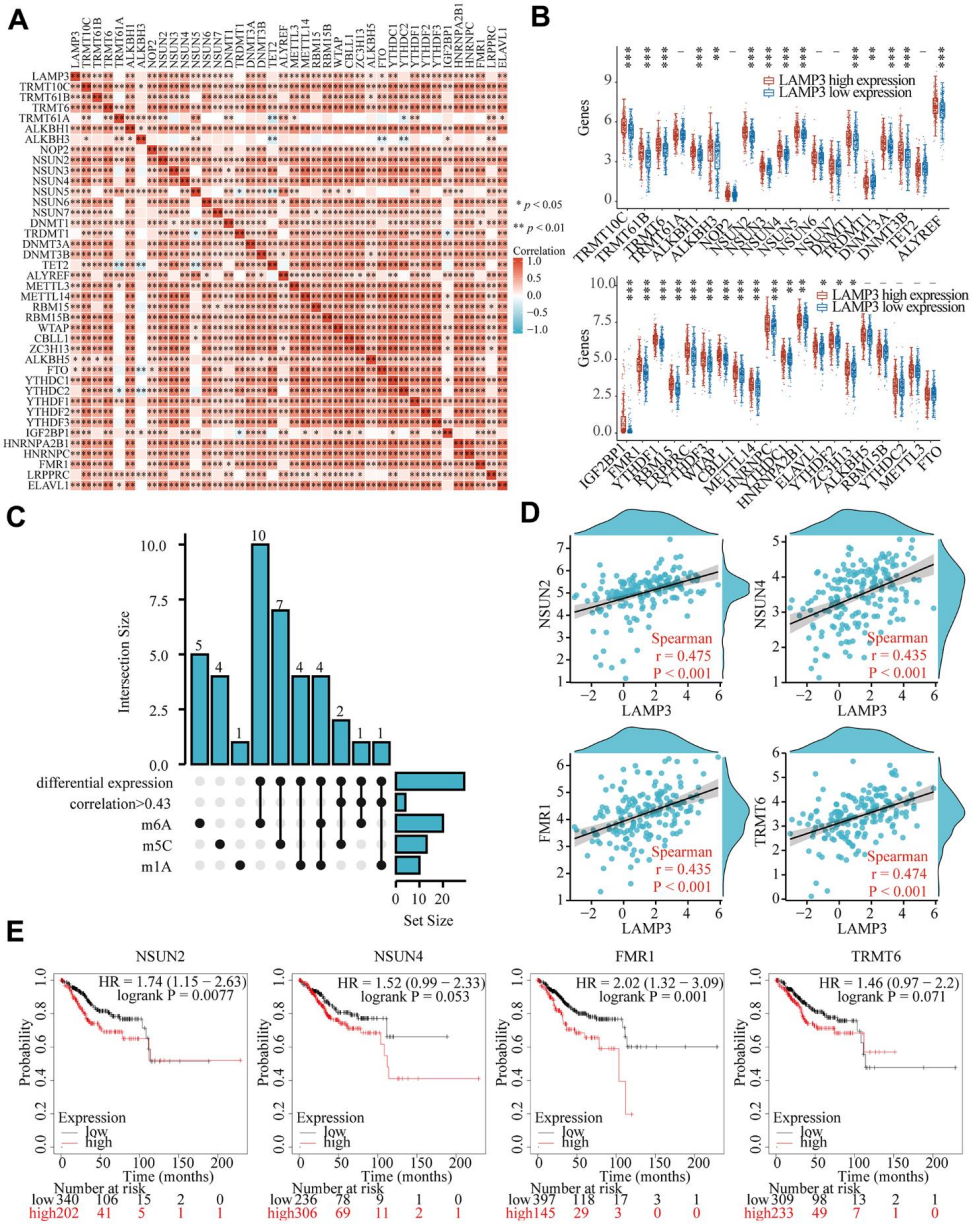
**Linkage of LAMP3 with RNA-modified genes in UCEC.** (**A**) Correlation of LAMP3 with various RNA-modified genes in UCEC. (**B**) Expression level of each of 39 genes between the high and low LAMP3 expression groups in UCEC. (**C**) The upset plot presents the genes that are individually screened under different conditions. (**D**) Association of NSUN2, NSUN4, FMR1, and TRMT6 with LAMP3 expression. (**E**) Effect of NSUN2, NSUN4, FMR1, and TRMT6 on the survival of UCEC patients.

An increasing number of reports confirm that RNA modifications have an essential modulatory role in tumor pathogenesis, which prompts us to study their link with LAMP3 in UCEC. To start with, by means of data from TCGA, we examined the connection of LAMP3 with 39 m6A, m5C, and m1A-related genes in UCEC, and the results displayed that 33 of these genes presented a very significant positive correlation with LAMP3 at the expression level (Figure 8A). In addition, grouping LAMP3 according to its expression level, we attempted to take an analysis of the differential expression level of these genes between the both groups with high and low LAMP3 expression in UCEC. As presented in Figure 8B, 29 of these genes conformed to our expectation, exhibiting differential expression. Subsequently, through upset plot, we selected 4 genes with correlation greater than 0.43 and consistent with differential expression, which were NSUN2, NSUN4, FMR1 and TRMT6 (Figure 8C). Scatter plots Figure 8D showed the molecular correlation specifically between LAMP3 and them. Furthermore, it was noteworthy that the Kaplan-Meier curves suggested that NSUN2, FMR1 may contributed to the poor prognosis of UCEC patients (Figure 8E). Altogether, these imply us that the way LAMP3 influences UCEC progression may have a connection with RNA modification.

### Gene - compound interaction network analysis

**Figure 9:**
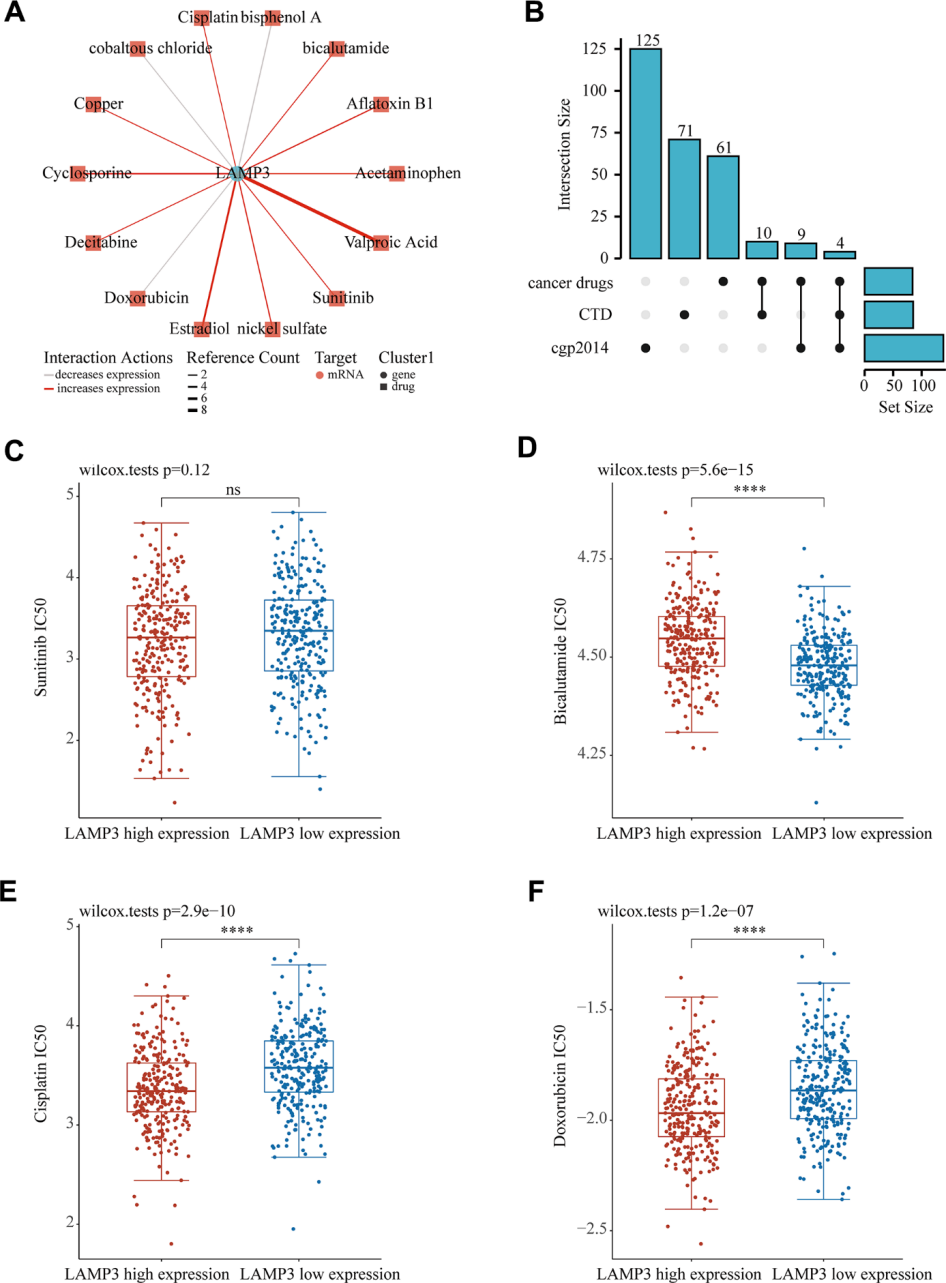
**Prediction of LAMP3 expression-related drugs.** (**A**) An advanced network diagram shows 14 cancer-related drugs that can modulate LAMP3 expression. (**B**) An upset diagram demonstrates drugs related to LAMP3 expression in CTD, cancer drugs and cgp2014. Relationship between LAMP3 expression and IC50 of (**C**) sunitinib (**D**) bicalutamide (**E**) cisplatin and (**F**) doxorubicin.

To predict some suitable compounds for the UCEC patients with high expression of LAMP3, we constructed a gene-compound interaction network. Through CTD, we identified 85 chemicals that can affect LAMP3 expression in UCEC at the mRNA level. Among these chemicals, we chose 14 drugs frequently treated for cancers to create an advanced network expression diagram, as illustrated in Figure 9A, where the red line represented a drug upregulating LAMP3 expression and the gray line doing the opposite. Subsequently, we intersected these drugs with cgp2014 and discovered 4 clocks of drugs, which were sunitinib, bicalutamide, cisplatin and doxorubicin (Figure 9B). Their efficacy was then evaluated by IC50 values, and it was found that patients with high LAMP3 expression were susceptible to cisplatin, doxorubicin, and resistant to bicalutamide (Figure 9C–9F). In short, these drugs have the potential to guide the treatment of UCEC patients with LAMP3 high expression.

### Interaction network and molecular docking model of LAMP3

**Figure 10:**
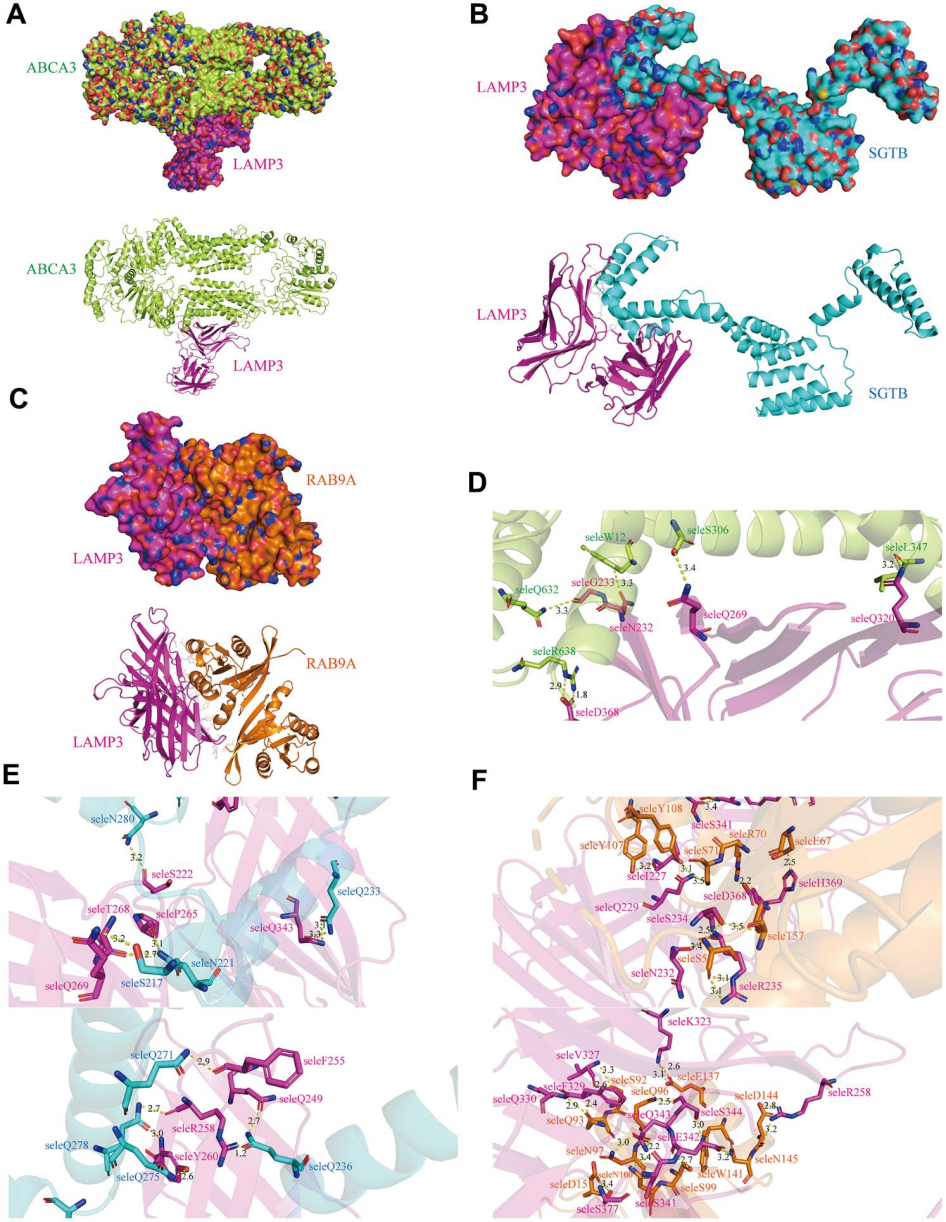
**Binding mode of LAMP3.** Cartoon view and surface view of (**A**) LAMP3-ABCA3 (**B**) LAMP3-SGTB (**C**) LAMP3-RAB9A molecular docking model, where LAMP3 is shown in pink. (**D**–**F**) Key regions that bind LAMP3 to ABCA3, SGTB and RAB9A, respectively.

As the most critical execution molecule, proteins often complete instructions by interacting with other proteins, hence, we deeply explored the interactions of LAMP3 with other proteins. At first, we built an interaction network of LAMP3 with 20 other proteins through the GeneMANIA online website, and it was surprising to note that LAMP3 interacted directly with three of them, namely ABCA3, SGTB and RAB9A (Supplementary Figure 6A). Following that, when we looked into the secondary structures of the four proteins, we noticed that ABCA3 possessed four structural domains and SGTB had two domains, while LAMP3 and RAB9A both contained only one structural domain, and these four proteins all had several modified sites (Supplementary Figure 6B–6E). Afterwards, with the outcomes being described in Figure 10A–10C, we examined the spatial structure of the four proteins from the PDB database, and predicted the molecular docking models of LAMP3 with ABCA3, SGTB and RAB9A by HDOCK server. In addition, we had also built hydrogen bonding networks for each of the three docking models, and the results revealed that there were 6 hydrogen bonds in the LAMP3-ABCA3 docking model,12 hydrogen bonds in the LAMP3-SGTB docking model, and only 26 hydrogen bonds in the LAMP3-RAB9A model (Figure 10D–10F).

### Construction of a ceRNA network involving LAMP3 in UCEC

It has been demonstrated that ceRNA networks function critically in various human cancers, thus we attempted to predict and build the ceRNA network of LAMP3 exploiting many biological information databases. First, we independently resolved 585, 136 and 16 target miRNAs of LAMP3 through TargetScan, DIANA and RNAinter databases individually, and the predicted findings were visualized by venn diagram. As presented in Supplementary Figure 7A, we could discover that 5 miRNAs were jointly predicted by these three tools. Moreover, we also probed the relevance of these miRNAs and LAMP3 at the expression level, with the discovery that 2 of them (has-miR-106a-5p and has-miR-499a-5p) were negatively regulated by LAMP3 (Supplementary Figure 7B).

Later, we applied miRNet and starBase databases to make predictions of lncRNAs potentially binding to has-miR-106a-5p and has-miR-499a-5p. As Supplementary Figure 7C displayed, the miRNet and starBase databases respectively predicted 72 and 79 lncRNAs targeting has-miR-106a-5p, with 32 lncRNAs situated in the overlapping part of the Venn diagram. However, there were only EBLN3P, H19, HAGLR, LINC02035, NORAD, SNHG14 consistent with the ceRNA network hypothesis in which lncRNAs were usually negatively correlated with miRNAs. As Supplementary Figure 7D indicated, miRNet and starBase databases predicted 27 and 25 lncRNAs targeting has-miR-499a-5p, separately, of which 14 were co-predicted, while only 1 matched the above negative correlation. With the above outcomes, we structured the Sankey diagram to illustrate the relationship between the predicted ceRNA networks (Supplementary Figure 7E).

## DISCUSSION

**Figure 11:**
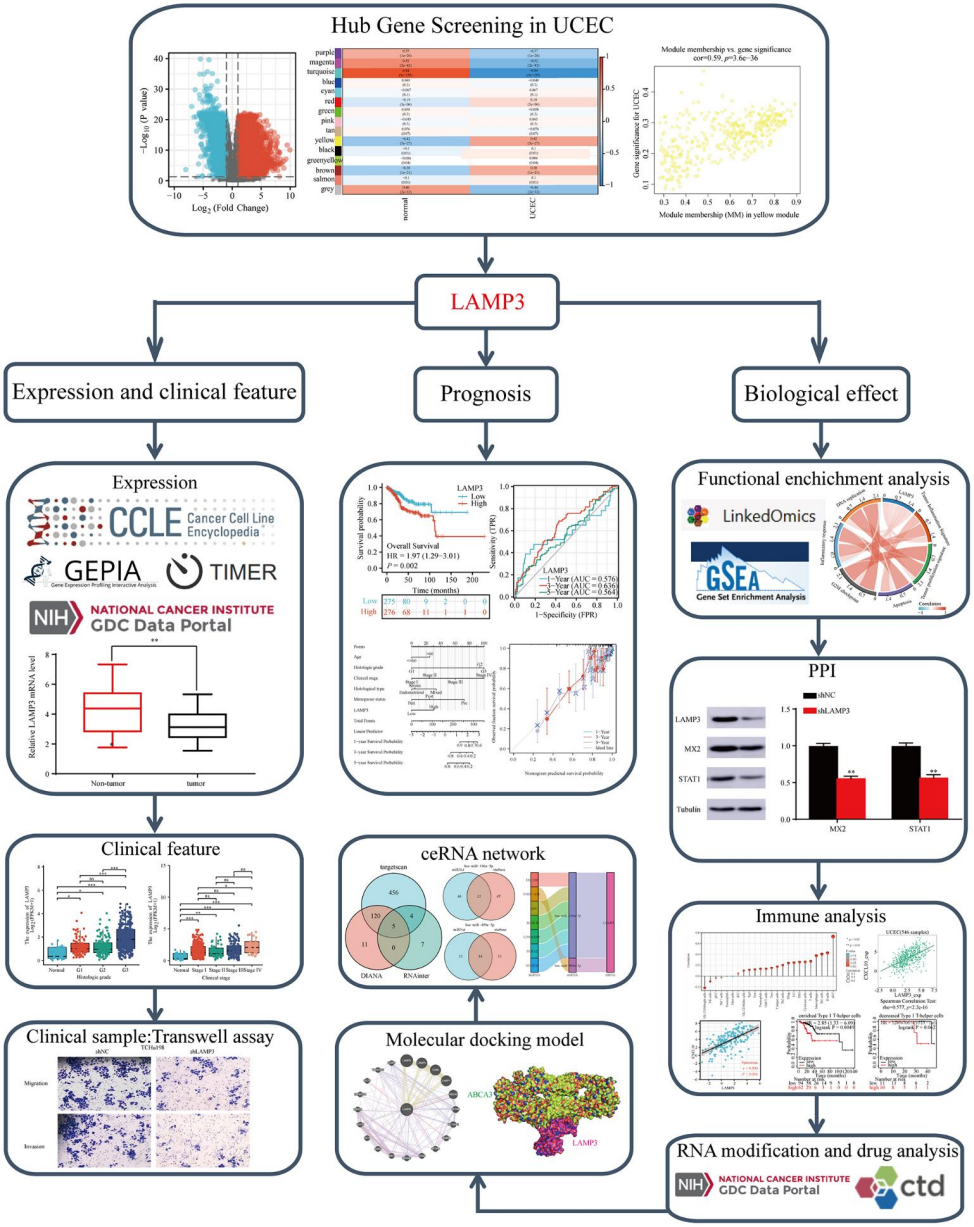
**Study workflow.** LAMP3 is a formidable biomarker for UCEC and could be a prospective candidate for the diagnosis, treatment and prognostic assessment of UCEC.

In this study, we used a combination of bioinformatics methods, functional analysis, and some experimental verification to screen for the key gene LAMP3 in UCEC and demonstrated its landscape in UCEC (Figure 11).

Endometrial cancer is amongst the most common gynaecological malignancies and poses a serious risk to women’s physical and mental health. In the last year, an estimated 66,570 new cases of endometrial cancer and 12,940 deaths related to endometrial cancer occurred worldwide [29]. Unfortunately, its incidence and mortality rates will keep rising due to lifestyle changes [30]. Previous studies have shown that abnormal expression of p53 and p16 can be used to identify high-risk endometrial cancer patients with poor prognosis, but this is still poorly used in clinical practice [31]; lower expression of ER and PR is associated with increased tumour-related mortality, but does not improve the prediction of individual mortality levels [32]. In recent years, studies on endometrial cancer have emerged thanks to the support of large sample databases, however, it is frustrating that clinical screening tests for UCEC are still not in place, and available biomarkers have not yet been applied to the clinic, so the purpose of our study was to integrate various bioinformatic analysis methods and utilize different online tools to characterize endometrial cancer from the perspective of multi-omics data. Targeting the characteristics of endometrial cancer to find more effective potential biomarkers to fill in certain gaps in previous studies. In this study, we assessed the expression and prognostic value of LAMP3 in UCEC based on bioinformatics and attempted to explain the possible mechanisms by which LAMP3 promotes the development of UCEC.

In this study, we analysed the presence of differentially expressed genes in UCEC by WGCNA and selected the most relevant gene set modules for cancer. Six genes, KLRG2, C5orf34, CCDC150, TDRKH, LAMP3 and KLC3, were selected after combining their prognostic value with the exclusion of existing studies related to UCEC, and each of these six genes was found to have a hazard ratio (HR) > 1 calculated by constructing a Cox model, indicating that exposure (high expression) is a risk factor for poor prognosis. Given the profound significance of LAMP3 in the progression of various gynaecological tumours including cervical, breast and ovarian cancers, this study focused on exploring the value of LAMP3 in UCEC.

Pan-cancer analysis showed that LAMP3 exhibits profound investigational significance in a wide range of cancers, with differential expression in up to 16 cancers, including upregulated expression in 12 tumour types. Subsequently, we examined LAMP3 overexpression in tumour tissues from both TCGA data and clinical samples. Moreover, LAMP3 can promote the invasion and migration of UCEC cells. We also found a correlation between LAMP3 expression and patient clinicopathological features previously. The expression profile of LAMP3 tended to be more upregulated with increasing tumour staging grade. Notably, deceased patients possessed higher expression levels compared to extant patients, driving us to delve into the underlying mechanisms that promote disease progression.

To achieve a broader insight into the potential mechanisms that LAMP3 may be involved in promoting tumourigenesis, we assessed its biological functions. In addition to its involvement in prominent cancer-related pathways such as the P53 pathway, PI3K/AKT/mTOR pathway and proliferation-related processes such as DNA replication and G2M checkpoint, we also noted that LAMP3 plays a particular and extensive role in immune-related basal responses, such as engagement in ANTIGEN_PROCESSING_AND_PRESENTATION, T/B_CELL_RECEPTOR_SIGNALING_PATHWAY, CYTOKINE_CYTOKINE_RECEPTOR_INTERACTION and other immune pathways that may mediate UCEC progression. Furthermore, the ability of LAMP3 to recruit Treg cells in bladder cancer and participate in the formation of an immunosuppressive microenvironment drove us to deepen our exploration of the specific immunological functions of LAMP3 in endometrial cancer [33].

There is mounting evidence that the occurrence, growth and metastasis of UCEC are very intertwined with the immune microenvironment [34–36]. LAMP3 expression has been reported to influence the prognosis of lung adenocarcinoma by regulating the infiltration levels of B cells, dendritic cells, natural killer cells, and eosinophils [37]. In the meanwhile, other studies have shown that the tertiary lymphoid structure gene signature of LAMP3 can be used for prognostic prediction of ovarian cancer [38]. Our study showed that high expression of LAMP3 elevated the StromalScore, ImmuneScore and ESTIMATEScore in endometrial cancer, and further studies showed that LAMP3 expression positively correlated with infiltration of various immune cells, including aDC, Th1 cells, B cells, Macrophages and Th2 cells. In contrast, LAMP3 expression was negatively correlated with infiltration of NK CD56 bright cells, pDC, and NK cells. We then selected immune cells with correlated expression and continued to investigate the effect of differential LAMP3 expression on their infiltration. It was seen that most of the immune cells with expression correlations had differences in infiltration, but not all of the immune cells with expression correlations had differences in infiltration, which could be ascribed to their low correlation with LAMP3 and a certain degree of differential LAMP3 expression was not sufficient to show differences in their infiltration levels. Chemokines promote the trafficking of immune cells during inflammation and play a crucial role in tumour-related immune responses. LAMP3 was found to be correlated with the expression levels of multiple chemokines, and not only that, LAMP3 was also closely associated with the expression profiles of multiple chemokine receptors, which are involved in immune cell infiltration from multiple perspectives. Interestingly, our previous work showed that high expression of LAMP3 corresponds to a poor prognosis in UCEC patients, whereas the results of the immune chapter suggest that the poor survival effects of this differential expression can be eliminated by decreasing Th1 cells, decreasing Th2 cells. Poorly, LAMP3 expression coincided with positive association with infiltration of both of these cells, which at the same time suggests us a potential mechanism by which LAMP3 affects patient outcome through immune response, but how LAMP3 regulates infiltration of Th1 cells, Th2 cells needs to be further investigated.

Immune checkpoints are a class of immunosuppressive molecules that regulate the degree of activation of the immune response and are involved in the regulation of the autoimmune response. However, this mechanism can also be mastered and hijacked by tumour cells to participate in the immune escape response. The overexpression rates of PD-1 (CD279) and PD-L1 (CD274) in endometrial cancer have been reported to be the highest of all gynaecological cancers, so we evaluated LAMP3 expression levels and the correlation of multiple immune checkpoints in detail. Unsurprisingly, LAMP3 showed a strong positive correlation with the expression levels of CD274. In general, PD-L1 expressed by endometrial cancer cells interacts with PD-1 expressed on activated B lymphocytes and T lymphocytes to limit the cytotoxic activity of lymphocytes and help tumour cells to participate in immune escape through a negative feedback system. This may partly explain the inability of tumour tissues with high LAMP3 expression to arrest the progression of tumour progression despite having highly infiltrated B cells and T cells. At the same time, LAMP3 expression was also closely associated with immune checkpoint molecules such as CXCL10, ICAM1 and BTN3A1, suggesting that it may be involved in multiple immune recognition signaling pathways mediating possible immune dysregulation. Thus, our study may provide some theoretical basis for immune checkpoint blockade (ICB) therapy.

Through the PPI network, we identified three proteins (ABCA3, RAB9A, SGTB) that have physical interactions with LAMP3 and predicted the construction of their docking scheme with LAMP3. Although it has been reported in the literature that ABCA3 can be processed for cleavage within LAMP3-positive vesicles, the role of LAMP3 is limited to acting as a marker for cells [39]. In parallel, RAB9A has been shown to activate the AKT/mTOR signalling pathway involved in cancer progression, as well as to inhibit apoptosis and promote invasion and migration of hepatocellular carcinoma cells [40]; SGTB can be regulated by multiple miRNAs thereby deregulating a negative mechanism for cancer progression [41, 42]. Unfortunately, their mechanisms and roles in UCEC are not yet transparent, therefore, it is hoped that our work will provide some basis for future studies on their interactions and possible mechanisms affecting tumour progression.

The regulation of transcriptome expression levels involves multi-molecular interactions between lncRNA, miRNA, and mRNA. Almost all known cancer cells possess miRNA control of gene expression [43]. lncRNAs can compete with mRNAs to bind miRNAs, namely sponge adsorption, which in turn derepresses miRNAs from their target genes and elevates target gene expression levels. In this study, two miRNAs targeting LAMP3 (has-miR-106a-5p and has-miR-499a-5p) and their corresponding lncRNAs were identified. has-miR-106a-5p and has-miR-499a-5p have been previously studied to target NPAS2 and VAV3 respectively to regulate the progression of UCEC [44, 45]. Here, we also predicted the upstream targeting lncRNAs of these two miRNAs to construct a ceRNA network regulatory model and gain a more systematic understanding of the potential mechanisms regulating LAMP3 expression.

In recent publications, LAMP3 has been included in prognostic models for UCEC patients based on inflammatory response-related genes (IRGs) [46]. However, these studies did not provide a thorough explanation of how LAMP3 affects the progression of UCEC within the model. In contrast, our study employed WGCNA and other methods to screen and investigate LAMP3. We briefly examined its expression across different types of cancer and specifically explored its role in UCEC. We constructed a ceRNA network for LAMP3 in UCEC to elucidate its regulation of gene expression. Furthermore, we identified key events involving LAMP3 in UCEC progression through GO, KEGG, and GSEA analysis. Additionally, we constructed a PPI network for LAMP3. Our study extensively elaborated on the role of LAMP3 in immune infiltration, chemokines and their receptors, immunosuppressants and stimulants, immune-infiltrating cells, and immune survival across several pages. Moreover, we attempted to explain the significance of LAMP3 in UCEC through the context of epigenetic RNA modifications. Additionally, we predicted and constructed a docking model of LAMP3 with other gene products to explore the potential mechanism of LAMP3 in UCEC. At the therapeutic level, we proposed several potentially available drugs that could guide UCEC patients with high LAMP3 expression. Finally, we experimentally verified the differential expression of LAMP3 in UCEC and normal tissues, its effect on tumor cell invasion and migration ability, and its effect on the expression of the MX2 and STAT1.

In summary, our study specifically addressed the value of LAMP3 in UCEC. LAMP3 exhibits elevated expression levels in UCEC relative to normal tissues, and this differential expression profile is closely linked to clinicopathological features, and patients with high LAMP3 expression are often associated with a shorter survival expectancy. In addition, the high expression of LAMP3 is modulated by the designated ceRNA network. LAMP3 performs a variety of biological functions in the development of UCEC, particularly in relation to immunity. It has been shown that LAMP3 can mediate the progression and even the outcome of UCEC development by regulating the level of immune infiltration, linking relevant chemokines and their receptors, immune enhancers, immunosuppressants and even MHC molecules. Finally, we also propose that cells with high expression of LAMP3 are sensitive to several drugs and predict a possible docking pattern of LAMP3 with proteins such as RAB9A.

However, we must acknowledge several constraints pertaining to the present research. Firstly, it primarily depends on online public databases and bioinformatics computational approaches. Further experimental investigations are indispensable to robustly corroborate the function of LAMP3 and its correlation with immune cell infiltration. Secondly, specific inhibitors aimed at LAMP3 are currently in the development phase, and their clinical relevance has not been explicitly established. As such, additional clinical examinations in a laboratory setting are crucial to authenticate their involvement in UCEC.

## Supplementary Material

Supplementary Figures
