# Supplementary material for: LAMP3 is a potent uterine corpus endometrial carcinoma prognostic biomarker associated with immune behavior
Source: Aging (Albany NY). 2024 Jan 11;16(1):714–45. doi: 10.18632/aging.205414 (PMC10817406; doi:10.18632/aging.205414)
Supplement: Supplementary Figures [file aging-16-205414-s001.pdf]

SUPPLEMENTARY FIGURES

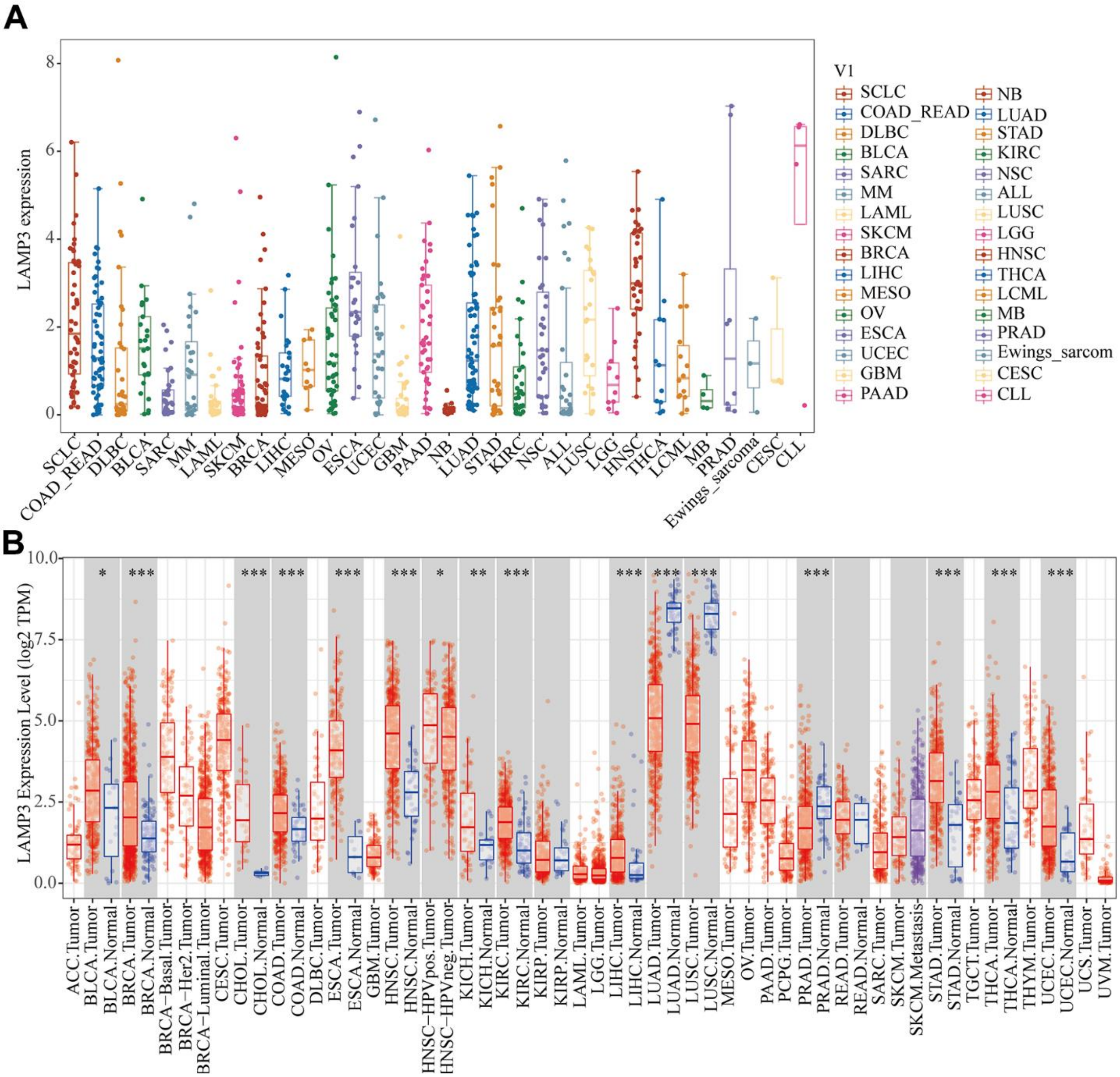

Supplementary Figure 1. (**A**) Expression distribution of LAMP3 in a variety of tumors in the CCLE database. (**B**) Expression of LAMP3 in normal and tumor tissues obtained from TCGA pan-cancer data from TIMER website.

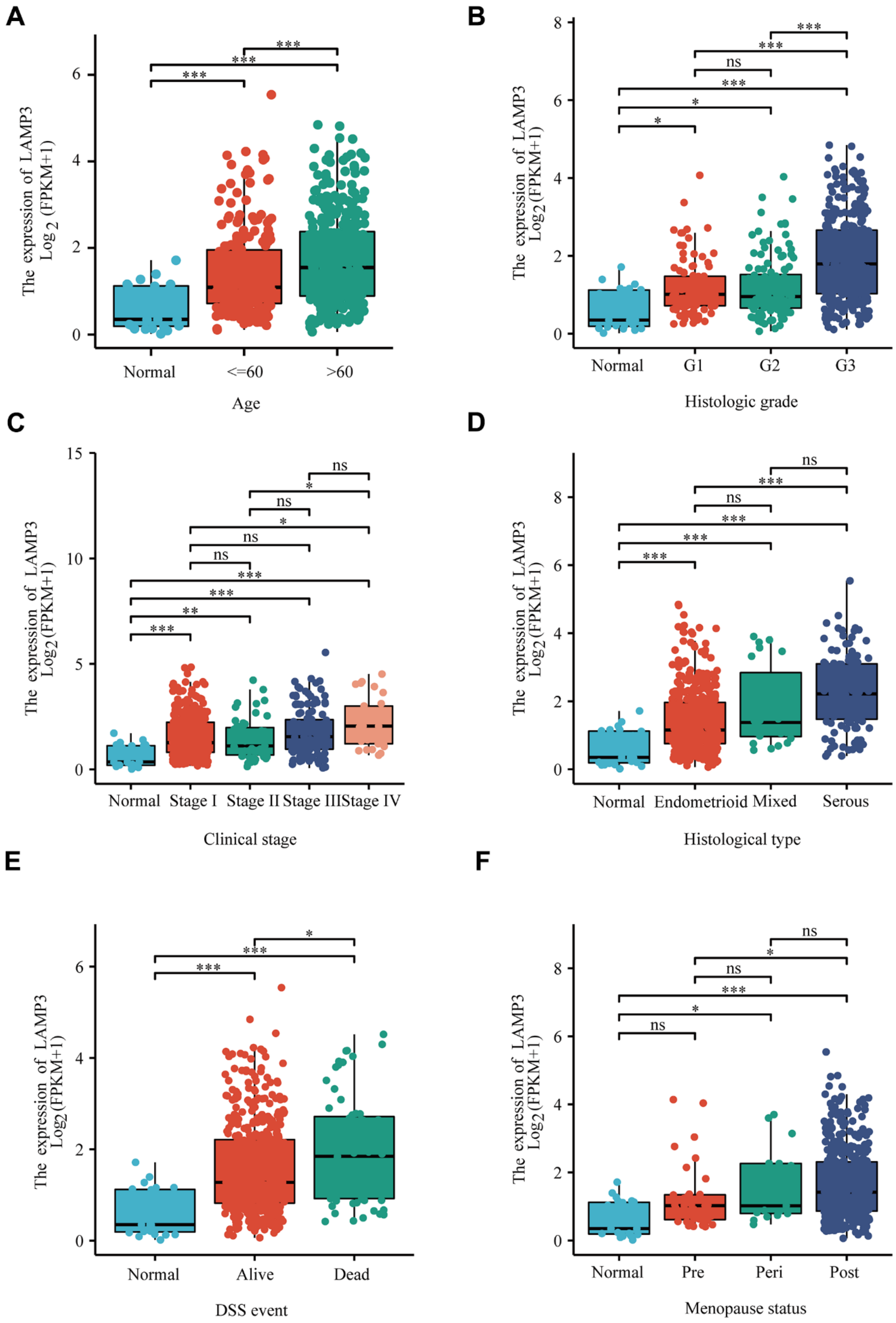

Supplementary Figure 2. LAMP3 expression is closely associated with various clinicopathological features. Box plots demonstrate the difference in LAMP3 expression among 6 subgroups according to (**A**) Age, (**B**) Histological grade, (**C**) Clinical stage, (**D**) Histological type, (**E**) DSS event, (**F**) Menopause status.

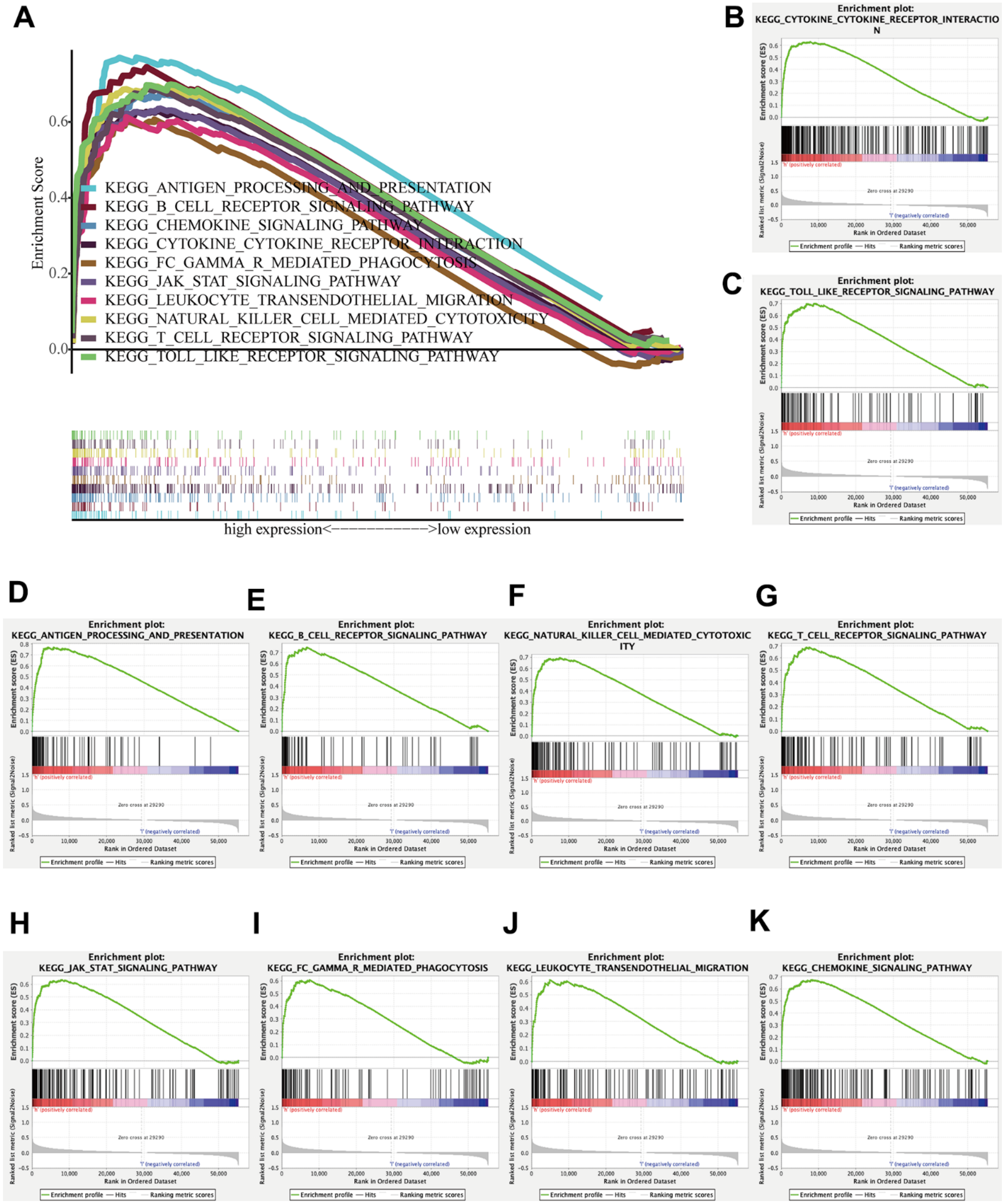

Supplementary Figure 3. (**A**–**K**) GSEA profiling reveals the enrichment pathways of LAMP3 high expression group.

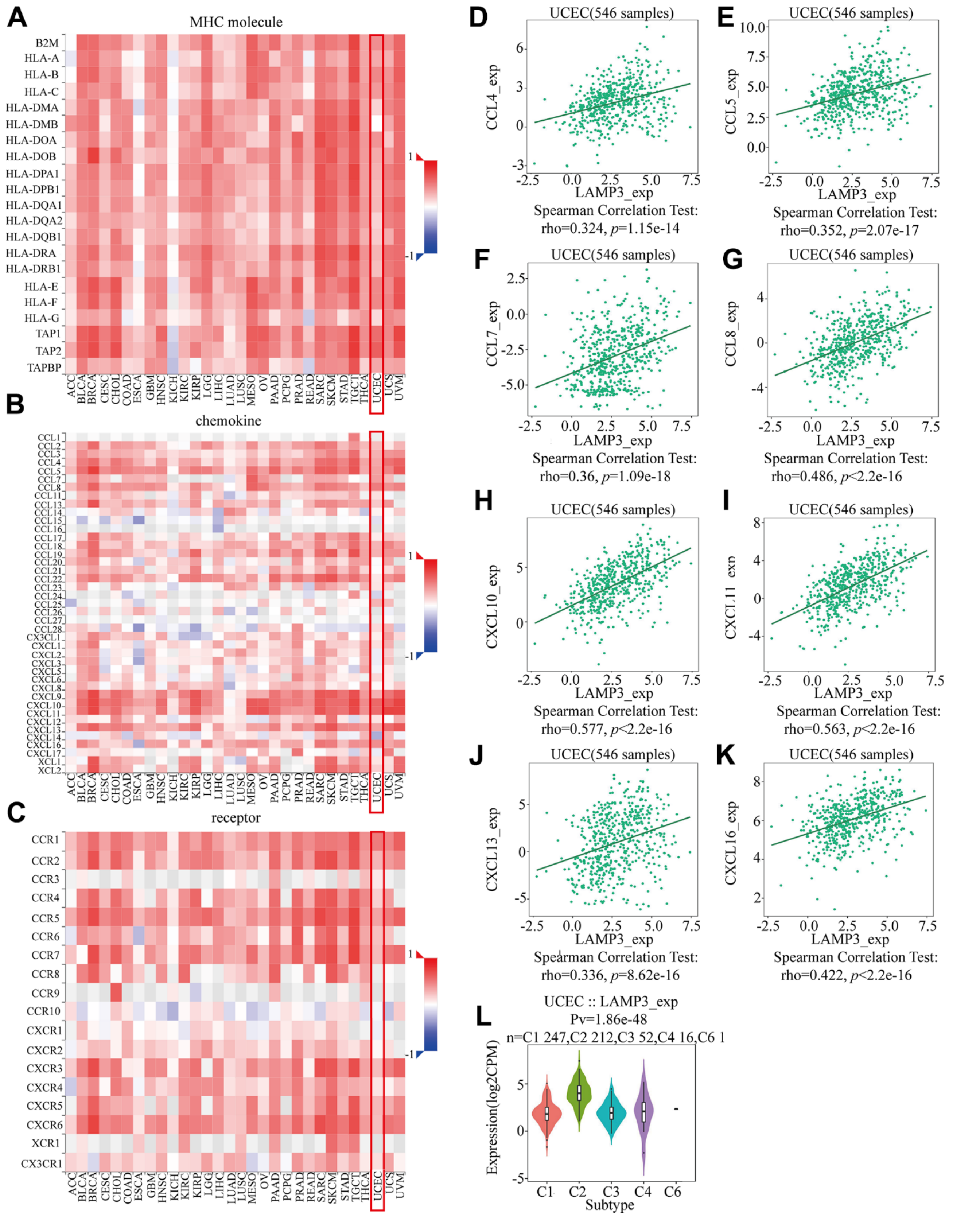

Supplementary Figure 4. Connection of LAMP3 with various cytokines in UCEC. Correlation of LAMP3 expression in pan-cancer with (**A**) MHC molecule, (**B**) chemokine, and (**C**) receptor. (**D**–**L**) Scatter plots of relationship between LAMP3 expression and CCL4, CCL5, CCL7, CCL8, CXCL10, CXCL11, CXCL13, CXCL16. (**E**) Expression level of LAMP3 among the 5 immune subgroups.

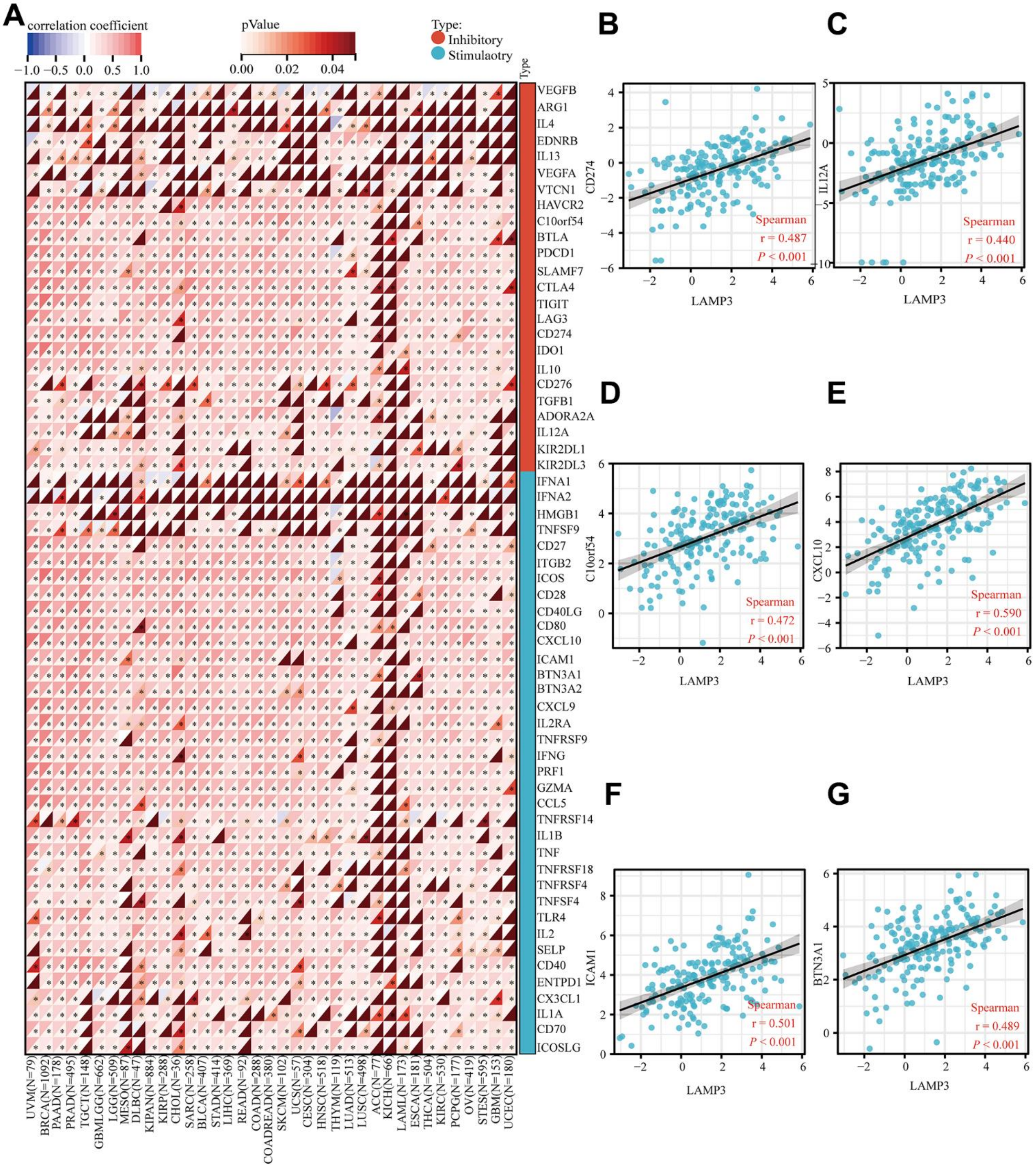

Supplementary Figure 5. Association of LAMP3 with various immune checkpoints in UCEC. (**A**) Heatmap illustrates the association of LAMP3 with immunostimulants and immunosuppressants in pan-cancer. (**B**–**G**) Scatter plots of the correlation between LAMP3 expression and CD274, IL12A, C10orf54, CXCL10, ICAM1, BTN3A1.

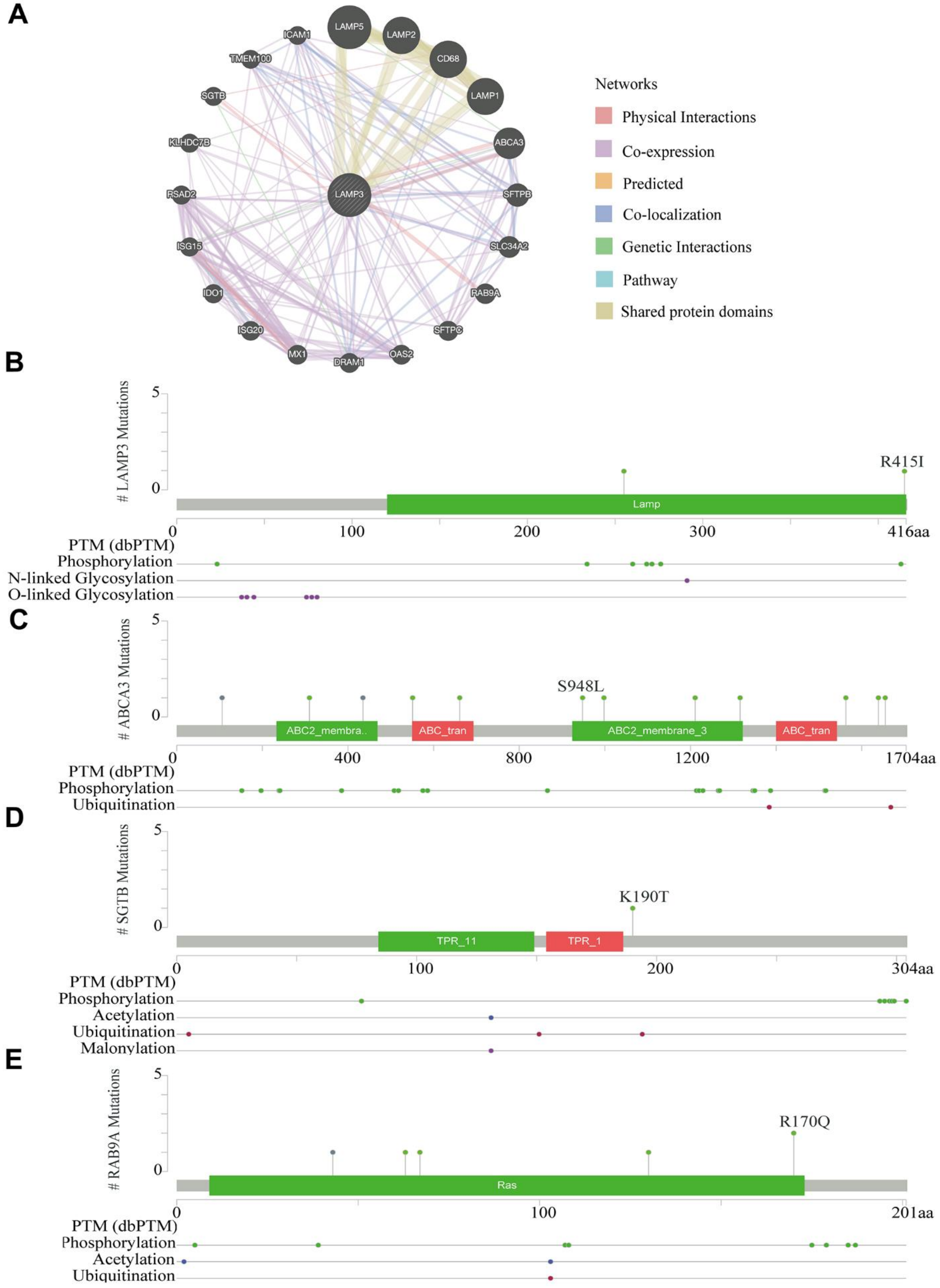

Supplementary Figure 6. Protein network secondary structure of LAMP3. (**A**) Interaction network of LAMP3 (different colored lines represent different roles). (**B**–**E**) Secondary structures of LAMP3, ABCA3, SGTB and RAB9A constructed by cBioPortal online website.

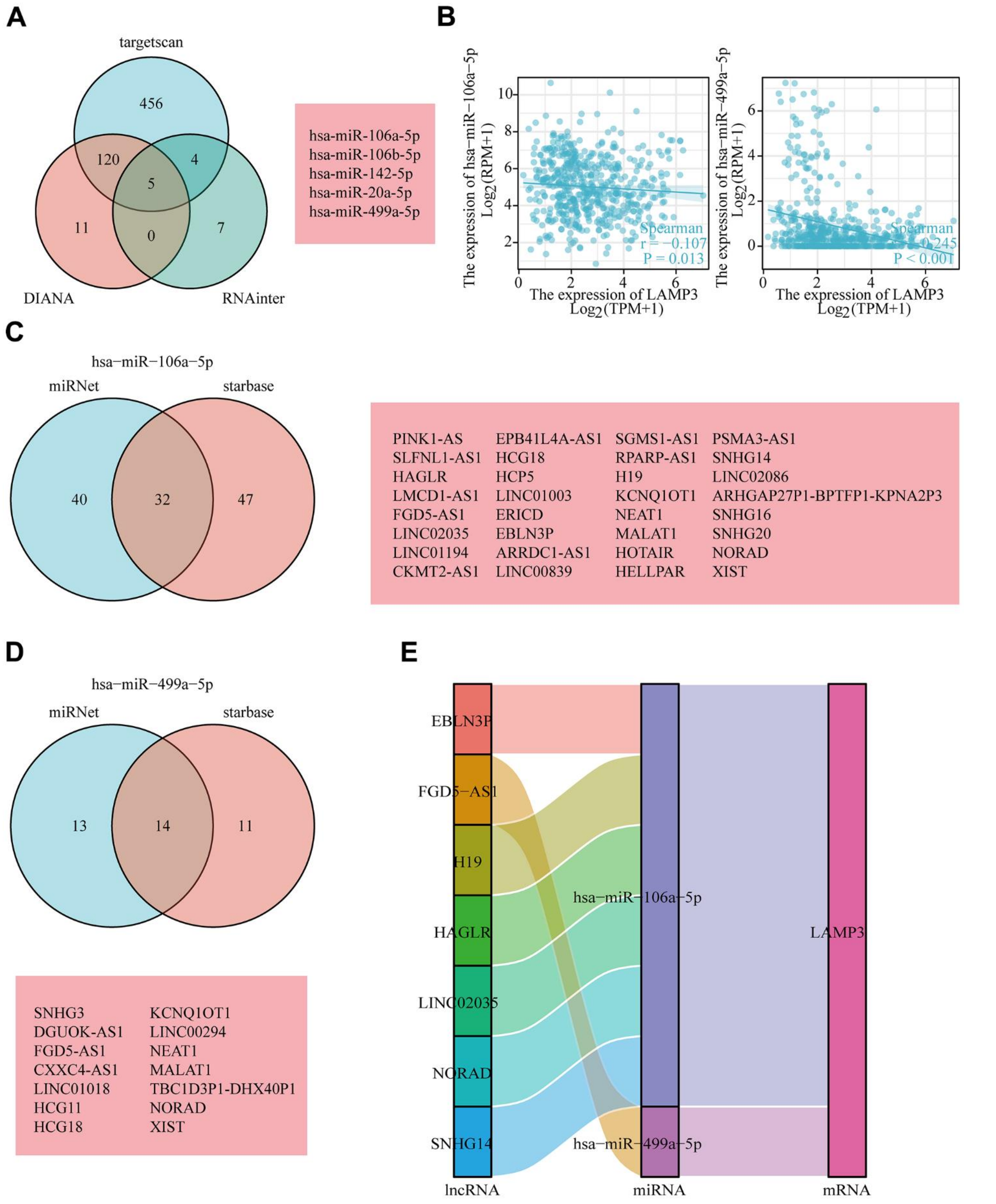

Supplementary Figure 7. Prediction and construction of lncRNA-miRNA-mRNA network of LAMP3. (**A**) Predicted miRNAs targeting LAMP3 in TargetScan, DIANA and RNAinter databases. (**B**) Correlation of has-miR-106a-5p and has-miR-499a-5p with LAMP3 expression. lncRNAs targeting (**C**) has-miR-106a-5p and (**D**) has-miR-499a-5p in miRNet and starBase databases. (**E**) Two pairs of ceRNA networks involving LAMP3.
